# Dual Bipolar Resistive Switching in Wafer‐Scalable 2D Perovskite Oxide Nanosheets‐Based Memristor

**DOI:** 10.1002/advs.202517588

**Published:** 2025-12-05

**Authors:** Sohwi Kim, Chansoo Yoon, Haena Yim, Taeyoon Kim, Hoyoung Suh, Woohyeon Ryu, Gwangtaek Oh, Jihoon Jeon, Kwanyoung Oh, Yeonjoo Jeong, Ji‐Won Choi, Bae Ho Park

**Affiliations:** ^1^ Division of Quantum Phases & Devices Department of Physics Konkuk University Seoul 05029 Republic of Korea; ^2^ Electronic and Hybrid Materials Research Center Korea Institute of Science and Technology Seoul 02792 Republic of Korea; ^3^ Center for Semiconductor Technology Korea Institute of Science and Technology Seoul 02792 Republic of Korea; ^4^ Advanced Analysis and Dater Center Korea Institute of Science and Technology Seoul 02792 Republic of Korea

**Keywords:** dual bipolar resistive switching, nanosheets, neuromorphic computing, remote supervised method, spike‐timing‐dependent plasticity

## Abstract

Memristors based on 2D materials are promising for compact and energy‐efficient neuromorphic hardware. However, conventional devices require paired elements to implement bidirectional weight updates, such as spike‐timing‐dependent plasticity (STDP) and anti‐STDP for supervised spiking neural networks (SNN) such as the remote supervised method. Here, an Au/Ti/2D Sr_2_Nb_3_O_10_ perovskite‐oxide nanosheet (SNO PON)/Pt memristor is demonstrated that exhibits dual bipolar resistive switching, supporting clockwise (interface) and counter‐clockwise (filament) switching. Ultrathin (≈5 nm) SNO PONs, fabricated over wafer‐scale areas by Langmuir–Blodgett deposition, serve as dynamic reservoirs for oxygen ions and vacancies. Voltage‐induced redox reactions at the Ti electrode are accompanied by the formation of oxygen vacancies in the SNO, as confirmed through cross‐sectional transmission electron microscopy and electron energy‐loss spectroscopy. The memristor exhibits stable resistance states with >10^3^ s retention and <0.2 V set variation across 30 cells. Bidirectional plasticity under dual‐polarity pulse trains replicates STDP/anti‐STDP rules, enabling a 3 × 3 array to encode pixel patterns with opposite‐polarity pulses. A leaky integrate‐and‐fire SNN model achieves 86.4 % accuracy on the MNIST dataset using identical pre‐ and post‐synaptic spike waveforms. These findings establish dual bipolar 2D memristors as scalable and efficient components for high‐density, simplified supervised SNN hardware.

## Graphical abstract

A wafer‐scalable memristor based on 2D Sr_2_Nb_3_O₁₀ perovskite oxide nanosheets exhibits dual bipolar resistive switching through controllable oxygen ion migration and redox reactions. This single device enables both STDP and anti‐STDP synaptic functions, achieving 86.4% MNIST accuracy in supervised spiking neural networks, offering a compact, energy‐efficient platform for neuromorphic computing.

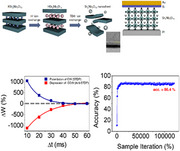

## 1 Introduction

2D material‐based memristors have the potential to revolutionize energy‐efficient computation by introducing multi‐state capabilities. The unique properties of 2D materials, such as edge, defect, and layered characteristics, can be precisely adjusted to enhance resistive switching (RS) behavior in memristors.^[^ ^1^, ^2^, ^3^ ^]^ This advancement paves the way for applications in brain‐inspired computing technology, such as a spiking neural network (SNN).^[^ ^4^, ^5^ ^]^SNN, often classified as a third‐generation artificial neural network (ANN), processes information through the utilization of the temporal arrangement of spikes.

Supervised learning, guided by instructional feedback, is widely regarded as a fundamental cognitive process inherent in the human brain, playing a crucial role in the acquisition of new knowledge and skills.^[^ ^6^ ^]^ The remote supervised method (ReSuMe) has been identified as a highly effective approach for supervised SNN.^[^ ^7^ ^]^ In the ReSuMe SNN model, the firing of both the remote “teacher neuron” and the “student neuron” is triggered by the input neuron. The weights linking these neurons are governed by spike‐timing‐dependent plasticity (STDP) and anti‐STDP learning rules, respectively. Note that only the weight changes (Δ*w*) of positive delay time (+ Δt) in the STDP and anti‐STDP learning rules are considered in ReSuMe since they are assumed to be zeros (Δ*w*  = 0) for the negative delay time (− Δt) in the STDP and anti‐STDP learning rules.^[^ ^8^ ^]^

According to the STDP learning rule in ReSuMe, when presynaptic spikes occur just before postsynaptic spikes with a positive delay time (+ Δt), the weight of the synaptic connection increases (potentiation). Conversely, the anti‐STDP learning rule dictates that, under the same conditions of pre and postsynaptic spikes with a positive delay time (+ Δt), the weight of the synaptic connection decreases (depression). To effectively implement both STDP and anti‐STDP characteristics for a positive delay time (+ Δt) within the ReSuMe algorithm, a pair of 2D material‐based memristors is typically required to accommodate the two types of RS directions: clockwise (CW) and counterclockwise (CCW). However, utilizing a pair of 2D material‐based memristors, each of which is dedicated to implementing either STDP or anti‐STDP, significantly increases the required footprint and complicates the circuit design or fabrication process. These challenges can be addressed by introducing a single 2D‐based memristor capable of exhibiting controllable CW and CCW RS behaviors, referred to as dual bipolar RS. This single memristor can implement both STDP and anti‐STDP, simplifying the design and reducing the complexity associated with traditional approaches.

Here, we demonstrate dual bipolar RS behaviors in Au/Ti/2D Sr_2_Nb_3_O_10_ (SNO) perovskite oxide nanosheet (PON)/Pt memristor, where ultrathin (5 nm) 2D SNO PONs serve as reservoirs for both oxygen vacancies and oxygen ions. Unlike conventional vapor‐phase deposited perovskite oxides grown by pulsed laser deposition or molecular beam epitaxy, the 2D SNO PONs used here are fabricated via liquid‐phase exfoliation and Langmuir–Blodgett stacking, forming atomically flat and strain‐free interfaces. This 2D assembly avoids the high‐temperature crystallization and interfacial intermixing typical of vapor‐grown films, thereby preserving the intrinsic layered structure and improving the reproducibility of bipolar switching.^[^ ^9^, ^10^ ^]^ The key property enabling controllable CW (interface‐type) and CCW (filamentary‐type) RS behaviors is the high mobility of oxygen ions and the ease of vacancy generation within the 2D SNO PONs. In this system, oxygen ions migrating or oxygen vacancies forming within the 2D SNO PONs create either an oxide layer or a conductive path, which supports the RS modes for CW or CCW, respectively. This dual bipolar RS capability allows the 2D SNO PONs‐based memristor to implement STDP and anti‐STDP within a single device using identical waveforms for supervised ReSuMe SNNs. This breakthrough offers a significant advancement toward overcoming the limitations of conventional von Neumann architectures and paves the way for compact, efficient neuromorphic hardware based on supervised SNNs through the unique properties of 2D material‐based memristors.

### 1.1 Fabrication and Properties of 2D SNO Perovskite Oxide Nanosheets

**Figure 1:**
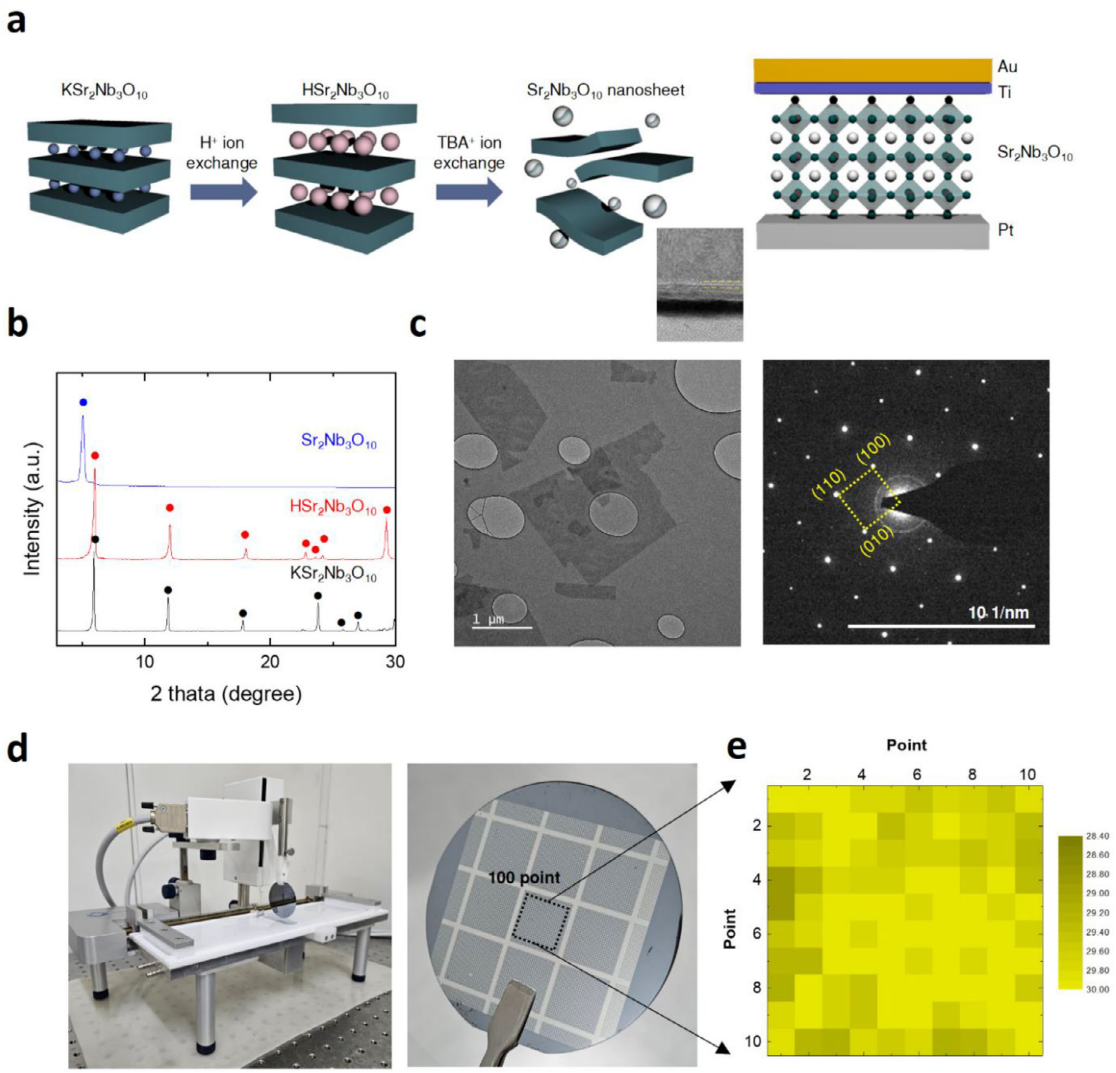
Wafer‐scale preparation and structural characterization of single‐crystalline Sr_2_Nb_3_O_10_ (SNO) perovskite oxide nanosheets (PONs). a) Schematic illustration of the Au/Ti/SNO/Pt on Si device stack and the corresponding fabrication process. b) Powder X‐ray diffraction (XRD) patterns of bulk KSNO (black), proton‐exchanged HSNO (blue), and exfoliated SNO nanosheets (red). c) Bright‐field transmission electron microscopy image of exfoliated SNO nanosheets (left; scale bar, 1 µm) and the corresponding selected‐area electron‐diffraction pattern (right). d) Photograph of a Pt/4‐inch Si wafer uniformly coated with a 5 nm‐thick SNO PON film. e) Spatial capacitance map measured at 100 positions across the wafer.

The SNO PONs‐based memristors, with an Au/Ti/5 nm SNO PONs/Pt/Si wafer structure, were fabricated as illustrated in **Figure** **1**a. The SNO PONs were synthesized using a two‐step cation exchange process to produce large‐scale 2D monolayers. Initially, KSr_2_Nb_3_O_10_ (KSNO) bulk ceramic, which has a layered perovskite structure, was synthesized via a solid‐state method. Next, the K^+^ ions in KSNO were substituted with H^+^ ions by stirring the compound in an HNO_3_ solution, resulting in the formation of HSr_2_Nb_3_O_10_. The H^+^ ions were then exchanged with tetrabutylammonium (TBA^+^) ions, which have a larger ionic radius, to obtain single‐crystalline SNO PONs in solution. Figure 1b shows the X‐ray diffraction (XRD) patterns of the products from each synthesis step. The XRD patterns of the synthesized KSNO powder match well with reference patterns, confirming the formation of KSNO as a Dion‐Jacobson type layered perovskite and verifying the correct crystal structure. Upon substitution of K⁺ with H⁺, a slight rightward shift in the (001) XRD peaks is observed, indicating successful ion exchange with H_3_O⁺ ions, which aligns with the expected solution‐based exchange process. The final XRD patterns for exfoliated SNO nanosheets reveal a d‐spacing of 1.6 nm, consistent with previous reports. Moreover, the exfoliated monolayer SNO PONs, placed onto a holey carbon grid for transmission electron microscopy (TEM) analysis, exhibit high crystallinity as evidenced by the TEM image and selected area electron diffraction (SAED) pattern in Figure 1c, further supporting the successful fabrication and structural integrity of the monolayer nanosheets.

The primary advantage of utilizing the nanosheet solution obtained through this solution‐based process lies in its capability for large‐area fabrication, addressing a significant bottleneck in producing single‐crystal 2D materials. Additionally, depositing these nanosheets via the Langmuir‐Blodgett technique enables the fabrication of wafer‐scale thin films, as shown in Figure 1d. To evaluate the uniformity of the deposited SNO PONs, capacitance measurements were conducted at 100 points across the wafer. Each capacitor in the measured sample exhibited a diameter of 50 µm and a pitch of 400 µm (Figure S1, Supporting Information). As shown in Figure 1e, the capacitance values demonstrate a standard deviation of only 0.37, confirming the consistency across the sample and supporting the effectiveness of this solution‐based fabrication method for producing uniform, large‐scale thin films.

### 1.2 Dual Bipolar RS of the 2D SNO Perovskite Oxide Nanosheets‐Based Memristor

**Figure 2:**
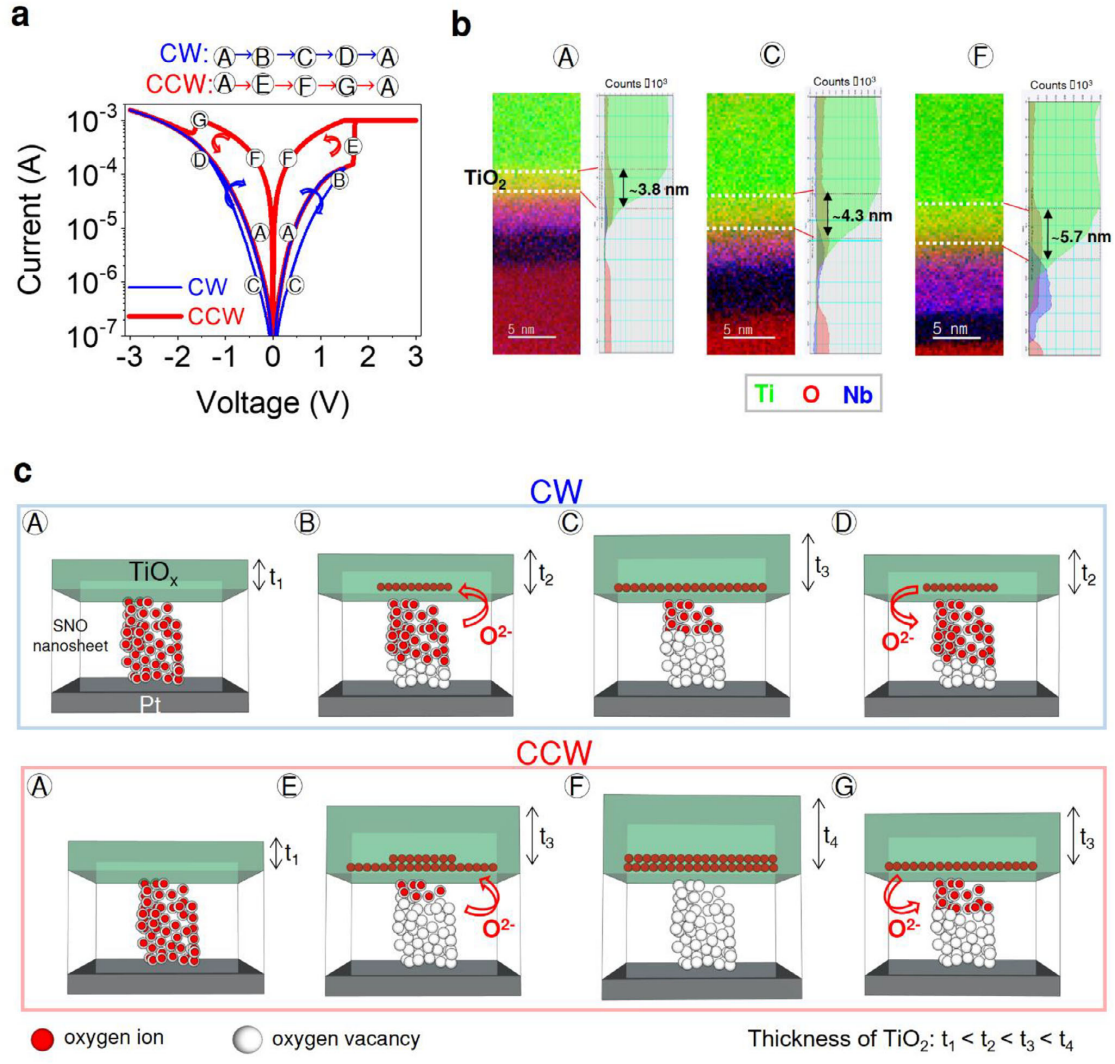
Dual bipolar resistive switching and its lattice‐scale origin in Au/Ti/2D SNO PONs/Pt memristor. a) DC current–voltage characteristics of a Au/Ti/2D SNO PONs/Pt memristor, showing both clockwise (CW, blue) and counterclockwise (CCW, red) switching loops. Labels A–G mark the characteristic states used in the mechanistic discussion. b) Cross‐sectional transmission electron microscopy (TEM) images and the corresponding electron energy‐loss spectroscopy (EELS) elemental maps at states A, C, and F. c) Atomistic model illustrating the proposed dual bipolar RS mechanism for the CW and CCW.

We characterized the dual bipolar RS behaviors in Au/Ti/ultrathin (≈5 nm) 2D SNO PON/Pt, as shown in **Figure** **2**a. Two distinct modes of RS with opposite switching directions are obtained. CW and CCW RS behaviors are identified as the blue and red current–voltage (*I*–*V*) curves, respectively. When the 2D SNO PON‐based memristor is in its initial state (“A”), a positive DC voltage applied to the top electrode initiates a CW RESET process (“B”), which switches the device to a high‐resistive state of the CW mode (HRS_CW_) (“C”). As the voltage sweeps into the negative regime, following the blue *I*–*V* curve in Figure 2a, a SET switching event occurs, transitioning the device to the low‐resistive state of the CW mode (LRS_CW_) (“D”). In summary, the blue cycle “A‐B‐C‐D‐A” in Figure 2a defines the CW RS mode, in which SET switching from HRS_CW_ to LRS_CW_ occurs under negative polarity, and RESET switching occurs under positive polarity.

Starting from the LRS_CW_ (“A”), applying a sufficiently large positive voltage (> *V*_*CW*, *RESET*_) triggers a CCW SET process, resulting in an abrupt transition (“E”) to the LRS of the CCW mode (LRS_CCW_) (“F”). Subsequently, applying a negative voltage with increasing amplitude initiates a CCW RESET process (“G”), moderately switching the device to the HRS of the CCW mode (HRS_CCW_). Following the red *I‐* *V* curve for a complete cycle, the sequence “A‐E‐F‐G‐A” defines the CCW RS mode, which operates with the opposite direction to the CW RS mode (blue loop). Retention measurements (see Figures S2c and S6c, Supporting Information) demonstrated that both CW and CCW resistance states remained stable for over 15 000 s, indicating the reliability and endurance of the dual bipolar RS behavior in the 2D SNO PON‐based memristor. We also demonstrate dual bipolar RS behavior in 30 individual 2D SNO PONs‐based memristors, confirming excellent device reproducibility (Figure S8, Supporting Information). The SET voltage distribution exhibits a mean of 1.84 V with a standard deviation of 0.19 V, indicating low device‐to‐device variability and uniform switching characteristics across the array.

Figure 2a illustrates the CW and CCW switching trajectories, though their lattice‐scale origins remain elusive. To elucidate the underlying mechanism, Figure 2b presents cross‐sectional transmission electron microscopy (TEM) images along with corresponding elemental maps obtained by electron energy‐loss spectroscopy (EELS) for the Au/Ti/2D SNO PONs/Pt memristor, quenched in distinct resistance states A, C, and F. The TiO_2_ interfacial layer increases in thickness from ≈3.8 nm (A) to 4.3 nm (C), and further to 5.7 nm (F), accompanied by a concurrent decrease in the O∕(Sn + O) ratio within the SNO nanosheet. The strong correlation between the growth of the interfacial TiO_2_ layer and oxygen depletion in the SNO nanosheet quantitatively verifies that Ti‐assisted redox reactions and vacancy generation occur in a cooperative manner. Although TEM provides localized information, all EELS analyses were performed under identical preparation and imaging conditions, and the consistent evolution of the Ti─L and O–K edge features confirms that the observed variations represent genuine state‐dependent redox behavior rather than spatial heterogeneity. These nanoscale observations provide direct experimental validation for the dual bipolar switching mechanism proposed in Figure 2c. Specifically, moderate oxygen extraction yields the HRS_CW_, whereas extensive vacancy alignment, along with thickening of the TiO_2_ layer, results in the LRS_CCW_.

Figure 2c presents a graphical illustration of the proposed model for the dual bipolar RS mechanism, which shares a common state (“A” in Figure 2a) observed in the 2D SNO PON memristor (see Notes S1 and S2, Supporting Information). The CW RS behavior is represented by the sequence “A‐B‐C‐D‐A” in Figure 2a, while the CCW RS behavior is characterized by the sequence “A‐E‐F‐G‐A” in Figure 2a. The complete switching scenario involves the coexistence of both CW and CCW RS behaviors, a shared initial state (“A”), and the two RS modes that are governed by the amplitude of positive voltage applied to the top electrode. This switching behavior is consistent with a self‐contained physical mechanism driven by key factors: the formation of oxygen vacancies within the oxide layer,^[^ ^11^, ^12^, ^13^ ^]^ and redox exchange reactions at the chemically active Ti electrode.^[^ ^14^, ^15^, ^16^ ^]^ Migration of oxygen ions creates oxygen vacancies within the SNO PON layer, which plays a crucial role in RS behavior. The chemically active Ti electrode facilitates exchange reactions with the migrated oxygen ions, further influencing the RS modes. These two mechanisms, oxygen vacancy formation and exchange reactions, work together to enable the dual bipolar RS behavior observed in the 2D SNO PON memristor, allowing for controlled transitions between CW and CCW modes depending on the applied voltage.

In the LRS_CW_ (“A”) of the CW RS mode, oxygen ions occupy their corresponding lattice sites, resulting in a nearly defect‐free configuration with a small density of uniformly distributed oxygen vacancies. When a positive external voltage smaller than the CCW SET threshold (i.e., *V* < *V*_*CCW*, *SET*_) is applied to the top electrode, oxygen ions migrate from the 2D SNO PON layer toward the Ti electrode and then undergo an exchange reaction with the chemically active Ti electrode (“B”). This process results in the formation of a thicker TiO_x_ layer (“C”) compared to that in state “A,” switching the memristor into the HRS_CW_ state. Under a subsequent negative external voltage, oxygen ions dissociated from the TiO_x_ layer migrate back into the 2D SNO PON layer and recombine with oxygen vacancies (“D”). This SET process in the CW RS mode reduces the TiO_x_ thickness, returning the memristor back to the LRS_CW_ state (“A”). Increasing the number of stacked 2D SNO PON layers from 5 to 10 results in a monotonic rise in the on/off ratio, consistent with the expansion of the Ti‐assisted redox region and the formation of a thicker interfacial TiO_x_ layer, as described in Figures S6 and S7 (Supporting Information).

On the other hand, the LRS_CW_ state (“A”) also corresponds to the HRS_CCW_ state (“A”) in the CCW RS mode. State “A” is not a perfectly vacancy‐free lattice but rather a metastable interfacial configuration containing a small yet finite concentration of uniformly distributed oxygen vacancies near the Ti/SNO interface. These vacancies enable both reversible interfacial modulation and localized redox activity depending on the intensity of applied bias. Under CW operation, they remain delocalized and participate in interfacial charge trapping and detrapping, giving rise to barrier‐controlled conduction (LRS_CW_). When a larger positive voltage (*V* > *V*_*CW*,*RESET*_) is applied to the top electrode, a stronger Ti–O exchange reaction occurs (“E”), and the local electric field becomes significantly enhanced at weak points pre‐existing in the SNO, driving field‐assisted drift and aggregation of these vacancies. This leads to the alignment of numerous oxygen vacancies within the 2D SNO PON layer, forming a conductive filament and switching the memristor into the LRS_CCW_ state (“F”). When a large negative external voltage is subsequently applied, oxygen ions stored in the TiO_x_ layer dissociate and migrate back toward the 2D SNO PON layer (“G”), where they recombine with the oxygen vacancies, leading to the rupture of the conductive path and switching the memristor back into the HRS_CCW_ (“A”).

To support the claim of interfacial/filamentary mechanisms, we analyzed the LRS current dependence on device areas. The area‐dependent current analysis (Figure S3, Supporting Information) clearly shows that the LRS current of the CW mode scales proportionally with the device area, whereas the CCW mode exhibits nearly area‐independent behavior, confirming that the CW and CCW switching originate from interfacial barrier‐limited and filamentary redox conduction, respectively. Additionally, to further distinguish the two mechanisms, space‐charge‐limited current (SCLC) fitting of the *I–V* curves was performed. The CW mode shows a progressive slope increase (*n* = 1.22, 2.24, and 3.02) typical of barrier‐limited and trap‐controlled conduction, whereas the CCW mode shows smaller exponents (*n* = 1.23–2.1), consistent with bulk‐limited filamentary behavior (Figure S11, Supporting Information). The electrical characteristics observed from the SCLC fitting are consistent with the structural evolution revealed by TEM/EELS, including the progressive thickening of the TiO_x_ layer and oxygen depletion in the SNO nanosheet.

Furthermore, during alternating CW↔CCW operation (150 cycles), the four read states are reproducible with σ/µ of 30–46%. While such variability reflects the stochastic nature of filament/interfacial processes in oxide memristors, state separability remains robust at 0.2 V (Figure S9, Supporting Information). Moreover, to confirm the material reliability of the 2D SNO PONs memristor under humid conditions, we performed humidity‐dependent measurements of the 2D SNO PONs. The *I–V* characteristics of the Au/Ti/2D SNO PONs/Pt memristor remain unchanged after 24 h of exposure to 90 % relative humidity at room temperature, confirming that the device retains its switching behavior and structural integrity under humid conditions (Figure S10, Supporting Information).

### 1.3 Two Opposite‐Directional Pulse Responses of the 2D SNO Perovskite Oxide Nanosheets‐Based Memristor

To implement STDP and anti‐STDP in a single memristor for advanced ReSuMe learning, which has advantages of a simplified fabrication process, reduced circuit complexity, and a smaller footprint, the memristor is required to show current responses in opposite directions under a pulse train with two polarities. Conventionally, memristors based on 2D materials exhibit controllable and reconfigurable synaptic functions achieved via external gating,^[^ ^17^ ^]^ or exhibit unidirectional current responses, typically showing either potentiation or depression under a pulse train of a single polarity (e.g., a (+) ‐ (‐) pulse pair for a potentiation‐depression curve).^[^ ^18^, ^19^ ^]^

**Figure 3:**
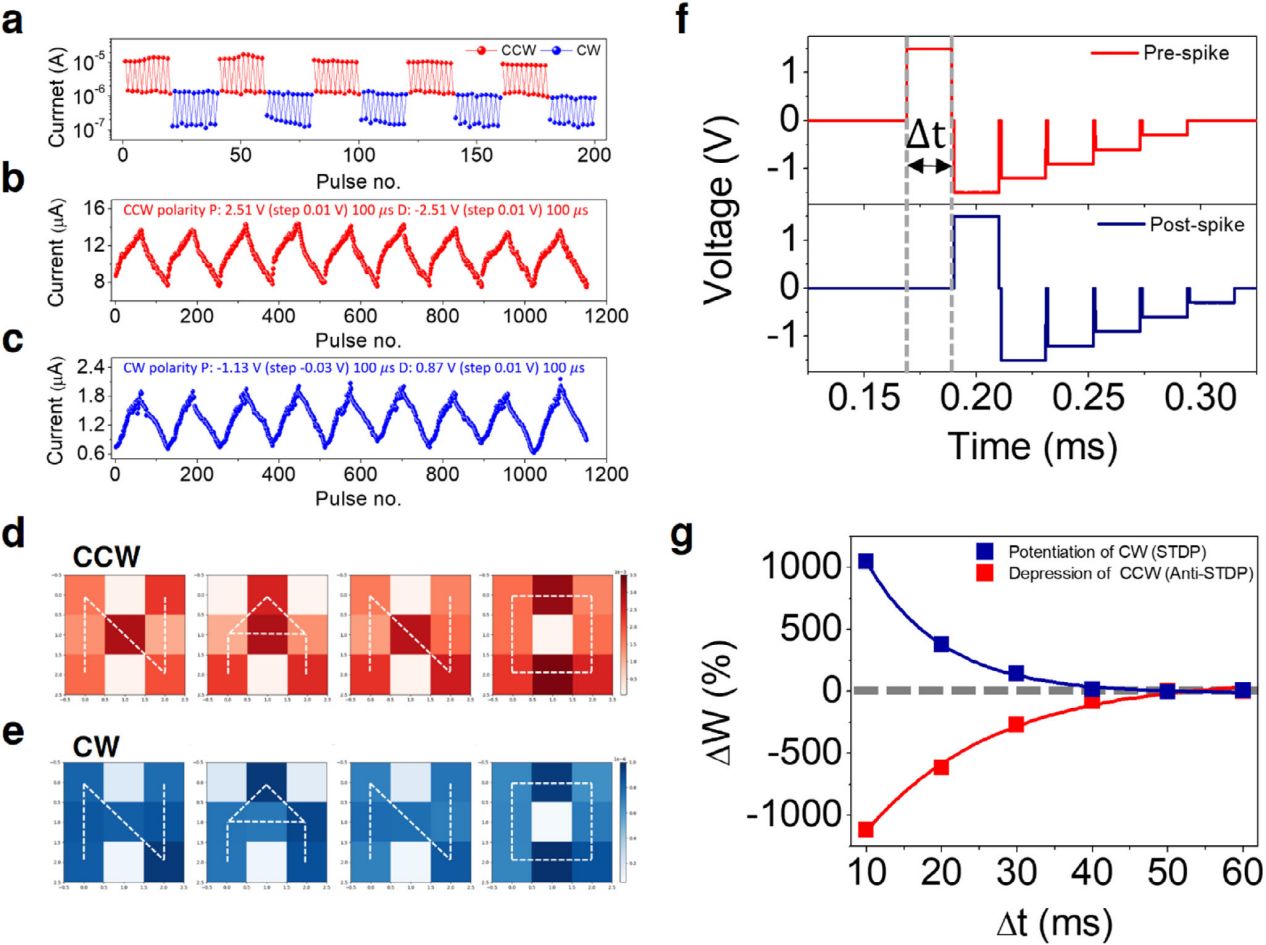
Pulse‐mode operation enabling STDP and anti‐STDP in (a) Au/Ti/2D SNO PONs/Pt memristor. a) Transient current responses under dual‐polarity pulse trains (20 ms width), illustrating CCW set/reset (3 V/–3 V, red) and CW set/reset (–3 V/1.5 V, blue) events in the same device. b,c) Potentiation–depression (P–D) curves obtained with incremental pulse amplitudes (100 µs width) for CCW (b) and CW (c) operation. d,e) Optical micrographs and read‐out current maps of 3 × 3 device arrays after programming the letters “N,” “A,” “N,” and “O.” High‐conductance pixels represent ON states, selectively written by 3 V pulses in the CCW mode and −3 V pulses in the CW mode. Scale bars, 200 µm. f) Schematic of identical pre‐ and post‐spike waveforms (positive time delay, Δt > 0) used for ReSuMe learning. g) Experimentally measured synaptic weight change (Δw) as a function of Δt, revealing co‐existing spike‐timing‐dependent potentiation (STDP, blue) and depression (anti‐STDP, red) in a single device.

In contrast to gate‐controlled approaches, our two‐terminal 2D SNO PON‐based memristor intrinsically exhibits dual bipolar resistive switching and reversible STDP/anti‐STDP within a single junction, without external gate modulation. Pulse mode experiments have revealed the coexistence of current responses in two opposite directions, as displayed by the CCW and CW set‐reset current responses in **Figure** **3**a. For the CCW (CW) mode, the pulse train had amplitudes of 3 V (−3 V) and −3 V (1.5 V), with a duration of 20 ms for the set and reset processes, respectively. A read voltage of 0.2 V with a duration of 20 ms was used. The 2D SNO PON‐based memristor demonstrates bidirectional responses under dual‐polarity pulse conditions within a single junction. This self‐modulated bidirectional plasticity arises from the coexistence of interfacial (CW) and filamentary (CCW) mechanisms, which distinguish our work from previously reported 2D materials. Furthermore, this 2D SNO PONs memristor exhibited an ultralow energy consumption of ≈0.14 pJ per synaptic update, comparable to recently reported ferroelectric (≈0.36 pJ) and RRAM‐based (≈0.1 pJ) neuromorphic devices, confirming its high energy efficiency for low‐power operation (Figure S13, Supporting Information).

Figure 3b,c shows the potentiation‐depression (P‐D) curves, which were obtained by applying dual‐polarity pulse trains. The pulse trains had amplitudes ranging from 2.51 to 3.14 V with 10 mV steps for potentiation in CCW mode, and from −2.51 to −3.14 V with 10 mV steps for depression in CCW mode. For CW operation, the pulse amplitudes ranged from −1.13 to −3.02 V with 30 mV steps for potentiation, and from 0.87 to 1.5 V with 10 mV steps for depression. All pulses had a duration of 100 µs. As a result, CCW and CW P‐D curves were achieved by the corresponding dual‐polarity pulse trains.

Conventional 2D material‐based memristor arrays typically demonstrate pattern memory using (+) ‐ (−) pulse trains for set‐reset operations, respectively.^[^ ^20^ ^]^ However, this unidirectional response of the set‐reset current is not suitable for single‐memristor‐based supervised SNNs using the ReSuMe algorithm. To demonstrate dual bipolar RS in an array, we fabricated a 3  ×  3 array of 2D SNO PON‐based memristors (Figure S12, Supporting Information). Using this array, we encoded and stored simplified letters “N,” “A,” “N,” and “O” as 3  ×  3‐pixel images. Occupied and unoccupied pixels corresponded to memristors written by the corresponding pulses: 3 and −3 V (duration of 20 ms) for CCW mode, and −3 and 1.5 V (duration of 20) ms for CW mode, respectively. The stored letters were retrieved using a 0.2 V read voltage and identified based on the resulting current, as shown in Figure 3d,e. These results highlight the dual bipolar RS capability of the 2D SNO PON‐based memristor array under oppositely polarized pulse trains, demonstrating its potential for implementing both STDP and anti‐STDP in a single memristor for advanced ReSuMe learning.

In hardware‐based SNN, each neuron typically requires a dedicated pulse generator to effectively perform STDP.^[^ ^21^ ^]^ The need to tune distinct pulse waveforms increases the device count per neuron, leading to a proportional scaling of the overall hardware complexity with the number of neurons.^[^ ^21^ ^]^ Additionally, in the ReSuMe learning algorithm, it is assumed that Δ*w* is zero for a negative delay time (− Δt).^[^ ^8^ ^]^ Therefore, we employed identical waveforms with a positive delay time (+ Δt) to simultaneously implement both STDP and anti‐STDP.

We expected that the dual bipolar RS behavior of the 2D SNO PON‐based memristor, under dual‐polarity pulse trains, could enable the realization of both STDP and anti‐STDP within a single memristor using pre‐ and post‐spikes of identical waveform. The identical waveforms for STDP and anti‐STDP, respectively, correspond to the SET operation voltage region of CW and the RESET operation voltage region of CCW in *I–V* curve. Specifically, the RESET region of CW ((+) *V* < (+) *V*_*CCW*, *SET*_) is distinct from the SET region of CCW ((+) *V* > (+) *V*_*CCW*, *SET*_). However, the SET region of CW overlaps with the RESET region of CCW.

When pre‐ and post‐synaptic spikes with a positive delay time (+ Δt) are applied to the 2D SNO PON‐based memristor, their superposition produces a combined spike waveform (Figure 3f). If this resulting waveform momentarily exceeds the threshold switching voltage, Δ*w* is induced, corresponding to potentiation in CW mode for STDP or depression in CCW mode for anti‐STDP, depending on the sign of Δt. Figure 3g shows the experimentally measured Δ*w* in the 2D PON‐based memristor with a positive delay time (+ Δt) of spike waveform. The overall Δ*w* effectively demonstrates the associated STDP or anti‐STDP behaviors.

### 1.4 ReSuMe SNNs Simulation

**Figure 4:**
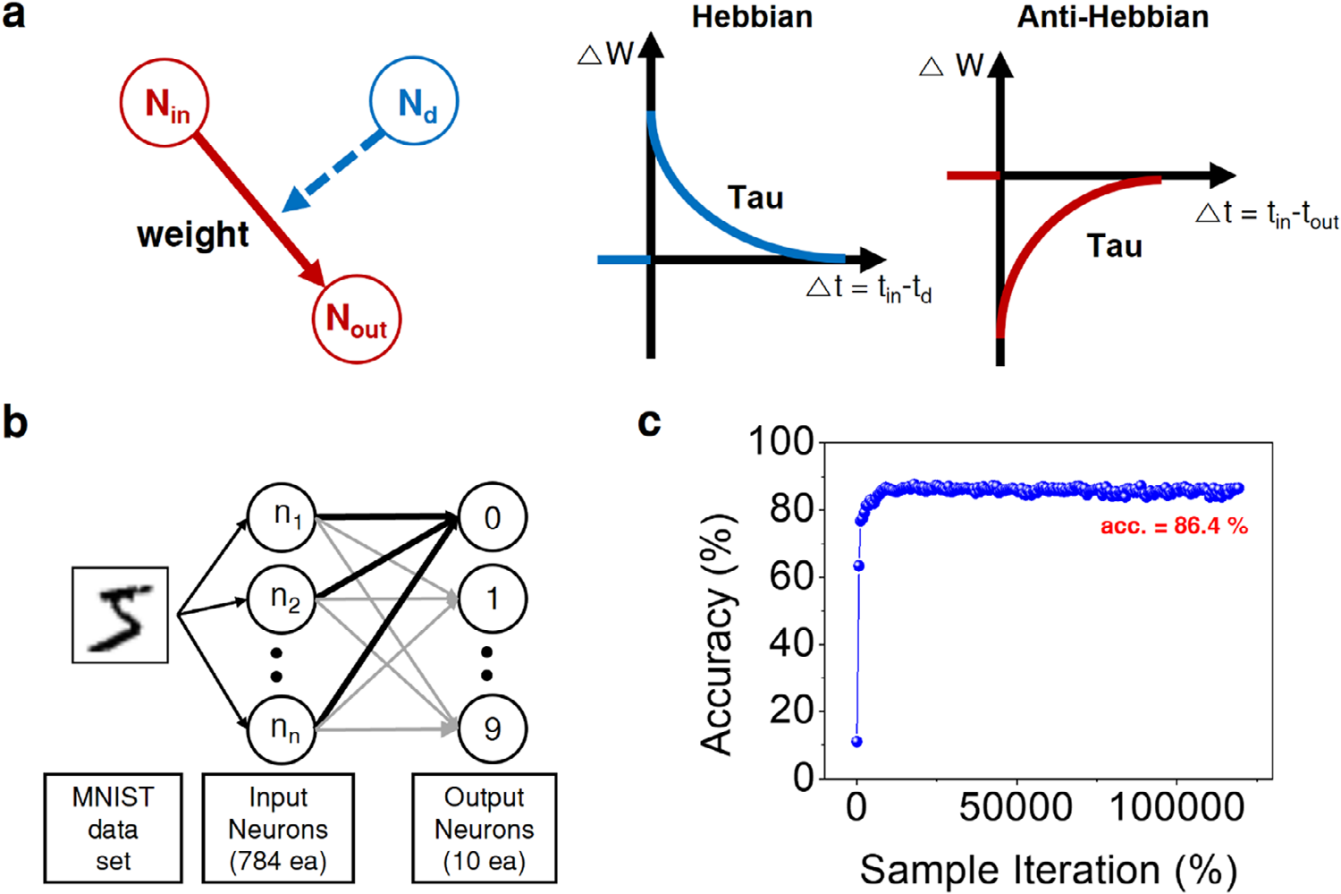
ReSuMe spiking‐neural‐network (SNN) simulation based on experimentally extracted device parameters. a) Synaptic weight‐update scheme in the ReSuMe algorithm: Hebbian potentiation occurs when a presynaptic spike precedes the target spike, while anti‐Hebbian depression is applied when a postsynaptic spike follows the presynaptic input. b) Network architecture employed for MNIST digit recognition, comprising 784 input neurons, 10 leaky integrate‐and‐fire output neurons, and synapses modelled using the Δw (Δt) characteristics from Figure 3g. c) Learning curve showing classification accuracy as a function of training iterations.

In the ReSuMe algorithm, synaptic weight updates are facilitated through two distinct mechanisms, as delineated in **Figure** **4**a. If the input from a presynaptic neuron precedes a target spike emitted by a teacher neuron, Hebbian learning is engaged to strengthen the synaptic connection. Conversely, if the postsynaptic neuron's spike follows the presynaptic input, anti‐Hebbian learning is employed to weaken the connection. This bidirectional adjustment of synaptic weights forms the core of the supervised learning process in the ReSuMe algorithm.

To evaluate the performance of the ReSuMe algorithm operating with a synaptic device based on a dual bipolar RS mechanism, we conducted an SNN simulation (Note S3, Supporting Information). This simulation employed a leaky integrate‐and‐fire (LIF) neuron model and was applied to the task of recognizing images from the Modified National Institute of Standards and Technology (MNIST) handwritten dataset. The network structure, depicted in Figure 4b, consists of 784 input neurons corresponding to the pixels in each MNIST image and 10 output neurons, matching the number of MNIST labels.

Based on the experimentally measured STDP characteristics, as depicted in Figure 3g, the discrete Δ*w*  −  Δ*t* data points were fitted to bi‐exponential functions representing potentiation (STDP) and depression (anti‐STDP) branches. The fitted model is given by:

(1) $$\Delta w=\left\{\begin{array}{c} A_{+}exp\left(-\frac{\Delta t}{\tau_{+}}\right),\;\;\;\;\;\;\;\;\;\;\;:for\;\;\;\;\;\;\;\;\;\;\;\;\;STDP\;\\ A_{-}exp\left(-\frac{\Delta t}{\tau_{-}}\right),\;\;\;\;\;\;\;\;\;\;\;:for\;anti-STDP \end{array}\right.$$

where A₊ and A₋ denote the maximum weight‐change amplitudes and τ₊ and τ₋ are the corresponding time constants. The parameters extracted from Figure 3g were A₊ = A₋ = 1.5, τ₊ = 10.083 ms, and τ₋ = 14.176 ms. These fitted functions were subsequently used in the ReSuMe‐based SNN simulation (Figure 4a–c).

To account for the device's finite conductance range and intrinsic non‐linearity, the resulting weight update (Δw) was mapped to the physical conductance window of g_min = 1 to g_max = 255 through exponential saturation functions implemented in the STDP and anti‐STDP routines within the python‐based simulator.

Using these fitted parameters, the SNN underwent 120 000 training iterations on the MNIST dataset and achieved a classification accuracy of 86.4%, demonstrating the effectiveness of the ReSuMe learning scheme when combined with the experimentally derived STDP behavior.

## 2 Conclusion

In summary, our demonstration of employing the same 2D SNO PONs‐based memristor to control both CW and CCW RS behaviors shows the feasibility of implementing both STDP and anti‐STDP characteristics within a single device. By effectively engineering the formation of oxygen vacancies and the migration of oxygen ions, we can induce filament‐type RS and interface‐type RS, respectively, providing a high degree of flexibility in controlling the polarity of the memristor's operation voltage. Consequently, the development of 2D SNO PONs‐based memristors not only simplifies the design but also reduces the complexity associated with conventional methods, minimizing the need for additional 2D material transfers and streamlining the fabrication process for ReSuMe‐based supervised SNN hardware.

## 3 Experimental Section

### Structural and Chemical Characterization

Transmission electron microscopy (TEM) was conducted using a Titan 80–300 (Thermo Fisher Scientific) operated at 300 kV to examine the microstructure and crystallinity. Electron energy‐loss spectroscopy (EELS) was performed using a Quantum 966 spectrometer (Gatan), integrated with the TEM, with an energy dispersion of 0.25 eV per channel.

### Device Fabrication

Single‐crystalline Sr_2_Nb_3_O_10_ nanosheets were deposited onto Pt (5 nm)/SiO_2_/Si substrates using the Langmuir–Blodgett technique. Subsequently, Au/Ti (30/25 nm) top electrode, patterned into 3 × 3 µm^2^ squares, was defined by electron‐beam lithography, followed by electron‐beam evaporation and lift‐off processes.

### Electrical Measurements

DC *I–V* characteristics, gradual current modulation, and spike‐timing‐dependent plasticity (STDP) under pulsed excitation were measured at room temperature in ambient conditions using a probe station equipped with a Keithley 4200‐SCS semiconductor characterization system and 2 µm W probes (MS TECH, M1.5BT).

## Conflict of Interest

The authors declare no conflict of interest

## Author Contributions

S.K., C.Y., H.Y., and T.K. equally contributed to this work and co‐wrote the manuscript. S.K., C.Y. performed the electrical measurements, and H.Y., H.S., K.O. fabricated the samples and carried out materials characterization. T.K. conducted the spiking neural‐network simulations. W.R., G.O., and J.J. reviewed the manuscript. Y.J., J.‐W.C., and B.H.P. conceived and supervised the project. All authors discussed the results and contributed to the final manuscript.

## Supporting information



Supporting Information

## Data Availability

The data that support the findings of this study are available on request from the corresponding author upon reasonable request.

## References

[1] B. Tang , H. Veluri , Y. Li , Z. G. Yu , M. Waqar , J. F. Leong , M. Sivan , E. Zamburg , Y.‐W. Zhang , J. Wang , A. V.‐Y. Thean , Nat. Commun. 2022, 13, 3037. 35650181 10.1038/s41467-022-30519-w PMC9160094

[2] M. Wang , S. Cai , C. Pan , C. Wang , X. Lian , Y. Zhuo , K. Xu , T. Cao , X. Pan , B. Wang , S. Liang , J. J. Yang , P. Wang , F. Miao , Nat. Electron. 2018, 1, 130.

[3] F. Hui , E. G. Gutierrez , S. Long , Q. Liu , A. K. Ott , A. C. Ferrari , M. Lanza , Adv. Electron. Mater. 2017, 3, 1600195.

[4] K. Zhu , S. Pazos , F. Aguirre , Y. Shen , Y. Yuan , W. Zheng , O. Alharbi , M. A. Villena , B. Fang , X. Li , A. Milozzi , M. Farronato , M. Muñoz‐Rojo , T. Wang , R. Li , H. Fariborzi , J. B. Roldan , G. Benstetter , X. Zhang , H. N. Alshareef , T. Grasser , H. Wu , D. Ielmini , Nature 2023, 618, 57. 36972685 10.1038/s41586-023-05973-1 PMC10232361

[5] U. Y. Won , Q. A. Vu , S. B. Park , M. H. Park , V. D. Do , H. J. Park , H. Yang , Y. H. Lee , W. J. Yu , Nat. Commun. 2023, 14, 3070. 37244897 10.1038/s41467-023-38667-3 PMC10224934

[6] C.‐C. Chang , P.‐C. Chen , B. Hudec , P.‐T. Liu , T.‐H. Hou , “Interchangeable Hebbian and Anti Hebbian STDP Applied to Supervised Learning in Spiking Neural Network,” IEEE International Electron Devices Meeting , IEEE, NewYork, 2018, 15.5.1–15.5.4.

[7] F. Ponulak , A. Kasiński , Neur. Comput. 2010, 22, 467. 10.1162/neco.2009.11-08-901 19842989

[8] Y. Zhou , N. Xu , B. Gao , F. Zhuge , Z. Tang , X. Deng , Y. Li , Y. He , X. Miao , IEEE Transact. Neural Netw. Learn. Syst. 2022, 33, 6640. 10.1109/TNNLS.2021.3082911 34081587

[9] A. Biswas , W. J. Kennedy , N. R. Glavin , P. M. Ajayan , J. Appl. Phys. 2023, 133, 240701.

[10] P. Santra , S. Ghaderzadeh , M. Ghorbani‐Asl , H.‐P. Komsa , E. Besley , A. V. Krasheninnikov , npj 2D Mater. Appl. 2021, 8, 33.

[11] I. Oh , J. Pyo , S. Kim , Nanomaterials 2022, 12, 2185. 35808021 10.3390/nano12132185 PMC9268157

[12] S. Pal , S. Bose , W. Ki , A. Islam , IEEE J. Electron Devices Soc. 2019, 7, 701.

[13] C. Hsu , T. Wang , C. Tsao , J. Alloys Compd. 2018, 769, 65.

[14] S. Asanuma , H. Akoh , H. Yamada , A. Sawa , Phys. Rev. B 2009, 80, 235113.

[15] K. Baek , S. Park , J. Park , Y.‐M. Kim , H. Hwang , S. H. Oh , Nanoscale 2017, 9, 582. 27886327 10.1039/c6nr06293h

[16] W. Kim , S. Menzel , D. J. Wouters , Y. Guo , J. Robertson , B. Roesgen , R. Waser , V. Rana , Nanoscale 2016, 8, 17774. 27523172 10.1039/c6nr03810g

[17] Cao, J. L. , T. Liu , W. Sang , Y. Gan , H. Li , Y. Wang , Z. Ren , Y. Yu , Z. Xin , Y. Chen , X. Zhang , D. Xiang , Q. Liu , Sci. Bull. 2025, 70, 3351. 10.1016/j.scib.2025.05.044 40555614

[18] P. Saha , M. S. E. , S. Sathyanarayana , B. Das , ACS Nano 2024, 18, 1137. 38127715 10.1021/acsnano.3c10775

[19] K. Ranganathan , M. Fiegenbaum‐Raz , A. Ismach , Adv. Funct. Mater. 2020, 30, 2005718.

[20] L. Sun , Y. Zhang , G. Han , G. Hwang , J. Jiang , B. Joo , K. Watanabe , T. Taniguchi , Y.‐M. Kim , W. J. Yu , B.‐S. Kong , R. Zhao , H. Yang , Nat. Commun. 2019, 10, 3161. 31320651 10.1038/s41467-019-11187-9 PMC6639341

[21] J. F. Leong , Z. Fang , M. Sivan , J. Pan , B. Tang , E. Zamburg , A. V.‐Y. Thean , Adv. Funct. Mater. 2023, 33, 2302949.

