# Supplementary material for: Dual Bipolar Resistive Switching in Wafer‐Scalable 2D Perovskite Oxide Nanosheets‐Based Memristor
Source: Adv Sci (Weinh). 2025 Dec 5;13(11):e17588. doi: 10.1002/advs.202517588 (PMC12931178; doi:10.1002/advs.202517588)
Supplement: Supplementary file 1 — Supporting Information [file ADVS-13-e17588-s001.docx]

Supporting Information

**Dual bipolar resistive switching in wafer-scalable two-dimensional perovskite oxide nanosheets-based memristor**

*Sohwi Kim, Chansoo Yoon, Haena Yim, Taeyoon Kim, Hoyoung Suh, Woohyeon Ryu, Gwangtaek Oh, Jihoon Jeon, Kwanyoung Oh, Yeonjoo Jeong*, Ji-Won Choi*, Bae Ho Park**


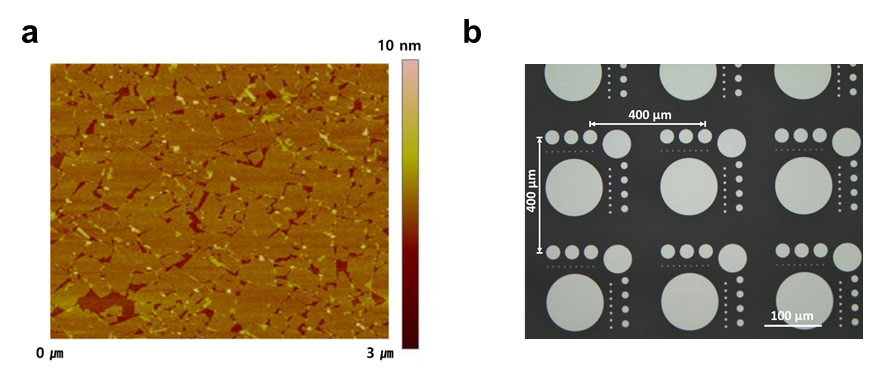


**Figure S1 | (a) AFM image of the nanosheet monolayer film after LB deposition. (b) Optical image of the circular top electrode array.**

Nanosheets were deposited via the Langmuir–Blodgett (LB) method and AFM analysis confirmed the formation of a uniform monolayer film. Using the same deposition technique, films with the desired thickness were subsequently fabricated. For capacitance measurements, a top electrode was deposited on the films, with each top electrode having a diameter of 50 µm and a pitch of 400 µm.


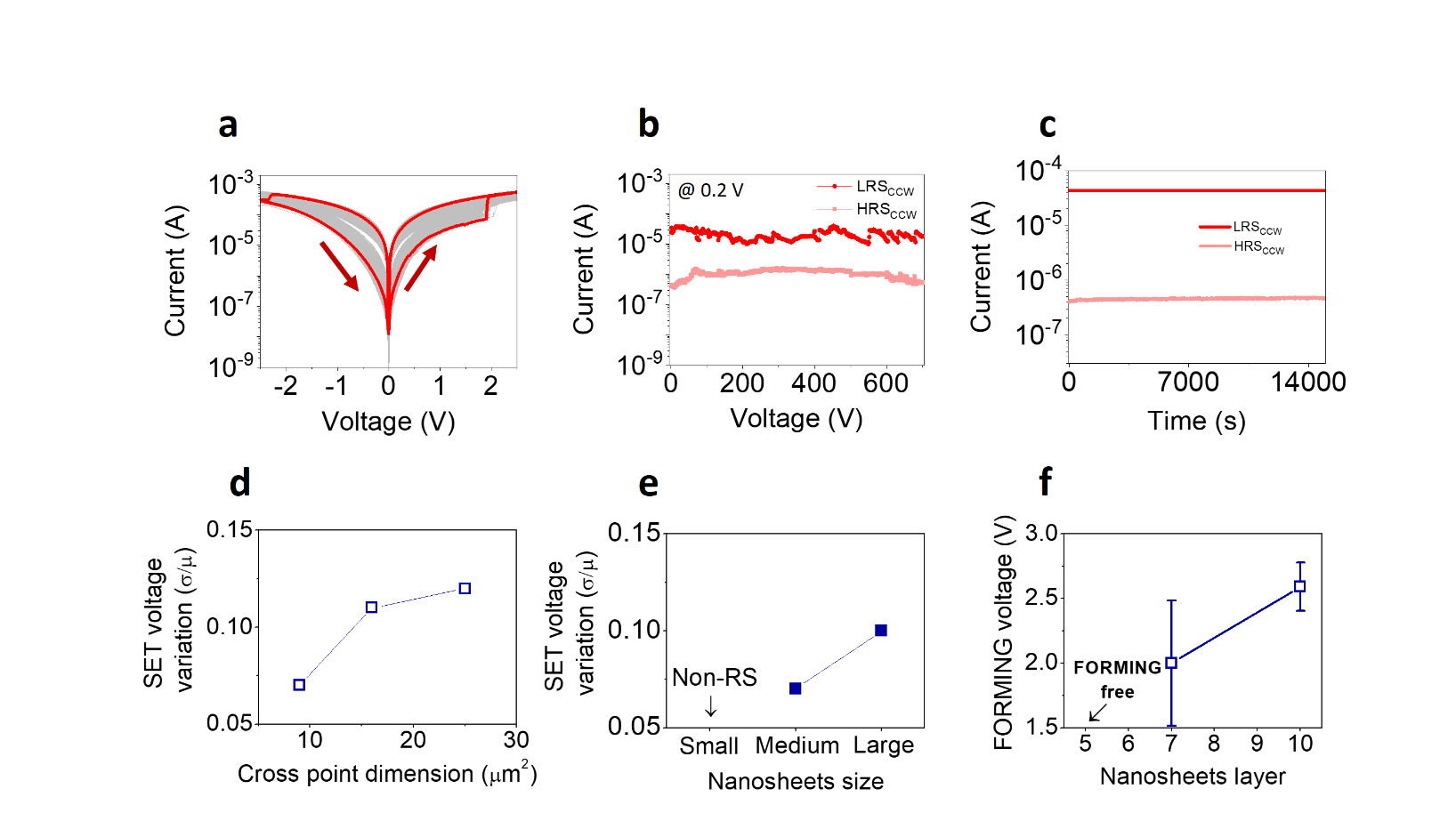
**Supporring Note 1**: CCW RS behaviors in Au/Ti/2D SNO PONs/Pt memristor

**Figure S2 | (a) *I-V* curves repeatedly obtained for 700 cycles. The blue arrows indicate the voltage sweep directions. (b) Current values for HRS and LRS measured for 700 cycles. (c) Retention test of multiple conductance states under varying compliance currents. Variation in the SET voltage as a function of (d) cross-point dimension of memristor and (e) the size of the 2D SNO PONs. (f) Relationship between forming voltage and the number of layers in the 2D SNO PONs.**

Figure S2a shows stable counterclockwise (CCW) resistive switching (RS) characteristics of the fabricated Au/Ti/2D Sr_2_Nb_3_O_10_ (SNO) perovskite oxide nanosheets (PONs)/Pt memristor over 700 cycles. We extracted high-resistive state (HRS_CCW_) and low-resistive state (LRS_CCW_) current values at a reading voltage 0.2 V from the *I-V* curves shown in Figure S2a. Figure S2b displays the current values of HRS_CCW_ and LRS_CCW_. The on/off ratio is defined as the ratio of current values at LRS_CCW_ and HRS_CCW_, which is approximately 20. The mean current values for HRS_CCW_ and LRS_CCW_ are $1.09\times{10}^{-6} A$ and $2.02\times{10}^{-5} A$, respectively. Moreover, the retention characteristics with multiple resistance states are shown in Figure S1c, demonstrating stable retention over 15,000 s for both LRS_CCW_ HRS_CCW_ states.

Considering that oxygen vacancies (V_O_) are confined to the edges of the 2D SNO PONs, a strong correlation between the cross-point dimension (2D SNO PONs size) of memristor and its corresponding RS behavior is expected. To validate this, memristors with varying cross-point dimensions and 2D SNO PONs sizes were fabricated. Their *I-V* characteristics are shown in Figure S3. We extract variability in the set voltage for different cross-point dimensions and 2D SNO PONs sizes from the repeatedly obtained *I-V* curves in Figure S3. Variability in set voltage is defined as the standard deviation ($\sigma$) divided by the mean value ($\mu$). The results are summarized in Figs. S2d and S2e, respectively. Shrinking the cross-point dimension leads to reduced variability in the set voltage, as shown in Figure S2d. Similarly, the size dependency of 2D SNO PONs (excluding small sizes that fail to implement the RS due to leakage pathways) follows the same trend as cross-point dimensions. These findings suggest that the cross-point dimension and nanosheet size plays crucial roles in reducing the variability of SET voltage. Smaller cross-point dimensions and nanosheet sizes are expected to exhibit more uniform average V_O_ percolation lengths in 2D SNO PONs-based memristors. Typically, switching voltage variability in nanosheet-based memristors depends on the uniformity of defect percolation paths^1^. Higher randomness in defect percolation paths deteriorates switching uniformity^1^.

Forming-free behavior can be induced by achieving smooth dielectric breakdown in memristors with ultrathin layer of 2D materials^1-5^. Typically, 2D memristors with an increased number of layers require a forming process^2,6^. Figure S5 shows the *I-V* curves of memristors with varying numbers of 2D SNO PONs layers. The corresponding forming voltages are summarized in Figure S2f. Notably, the 5-layer 2D SNO PONs-based memristor exhibits forming-free switching characteristics due to smooth dielectric breakdown, whereas devices with 7- and 10-layers require a forming process. The RS behavior observed in ultrathin 2D material memristors without forming is likely due to the facile propagation of native lattice disorders throughout the entire 2D stack^2^.


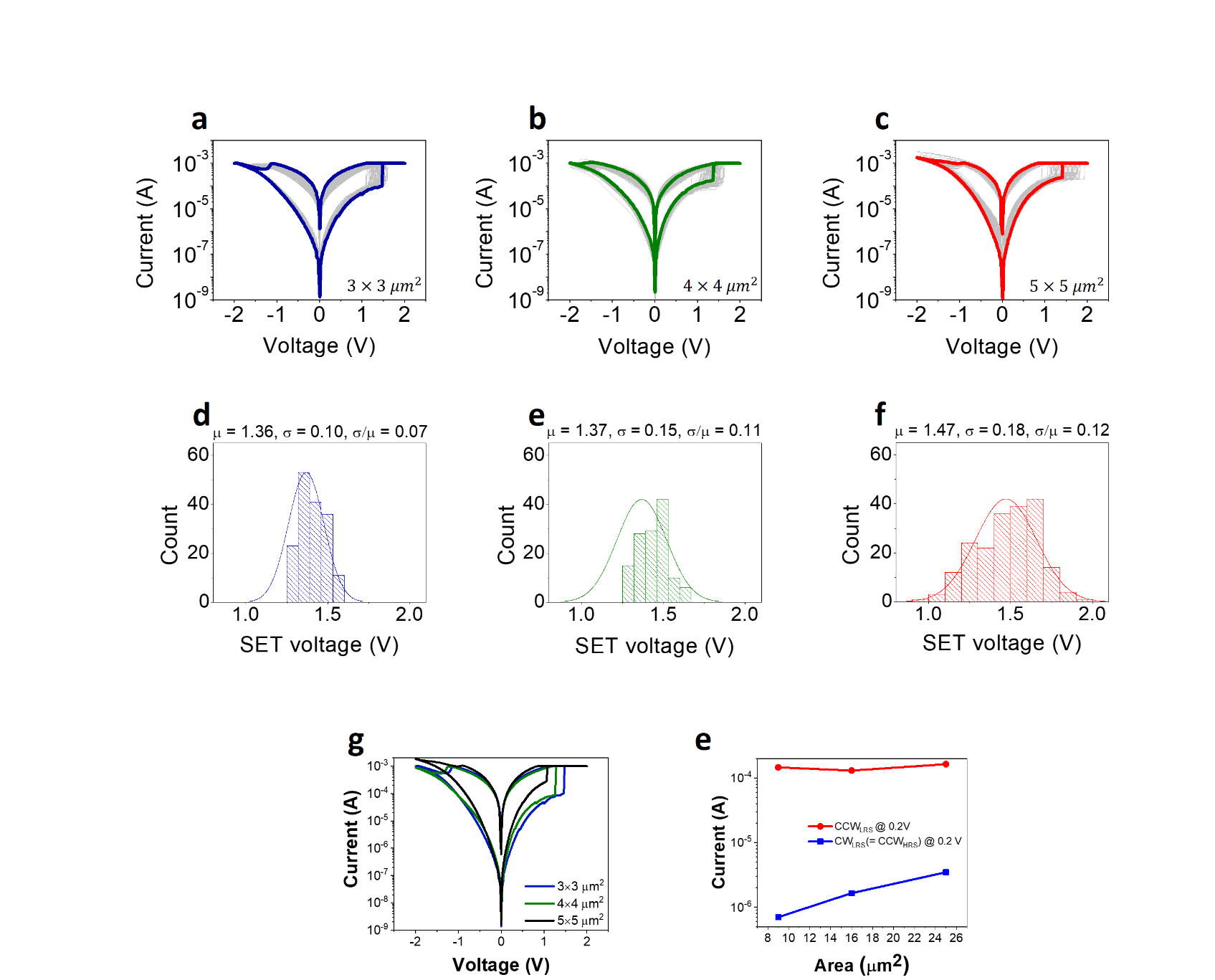


**Figure S3 | 200 representative *I-V* curves of 2D SNO PONs-based memristors fabricated with cross-point dimensions of (a)** $\boldsymbol{3\times3}$ **μm^2^, (b)** $\boldsymbol{4\times4}$ **μm^2^, and (c)** $\boldsymbol{5\times5}$ **μm^2^. Statistical analysis of the SET voltage for cross-point dimensions of (a)** $\boldsymbol{3\times3}$ **μm^2^, (b)** $\boldsymbol{4\times4}$ **μm^2^, and (c)** $\boldsymbol{5\times5}$ **μm^2^. Area dependence of (g) *I-V* curves and (e) LRS current states.**

For device areas of 3 × 3, 4 × 4, and 5 × 5 µm², the measured current at 0.2 V for the LRS_CW_ (= HRS_CCW_) increases nearly in proportion to the device area from 7.0 × 10⁻⁷ A (3 × 3 µm²) to 3.5 × 10⁻⁶ A (5 × 5 µm²), indicating uniform and area-scaled conduction dominated by interfacial barrier modulation. This quasi-linear (area-dependent) scaling is commonly observed in interface-limited transport because local barrier inhomogeneity and interfacial microstructure induce deviations from ideal Ohmic area scaling^7^.

In contrast, the LRS_CCW_ current remains almost constant with increasing device area (1.45 × 10⁻⁴ A, 1.30 × 10⁻⁴ A, 1.63 × 10⁻⁴ A for 3×3, 4×4, and 5×5 µm², respectively), showing no significant area dependence. This behavior is characteristic of localized filamentary conduction, where the current path is confined to nanoscale filaments independent of the electrode area.

As a result, these analyses quantitatively confirm that the CW and CCW modes are governed by two distinct switching mechanisms: an interfacial barrier-modulation process for CW and a filamentary redox process for CCW.


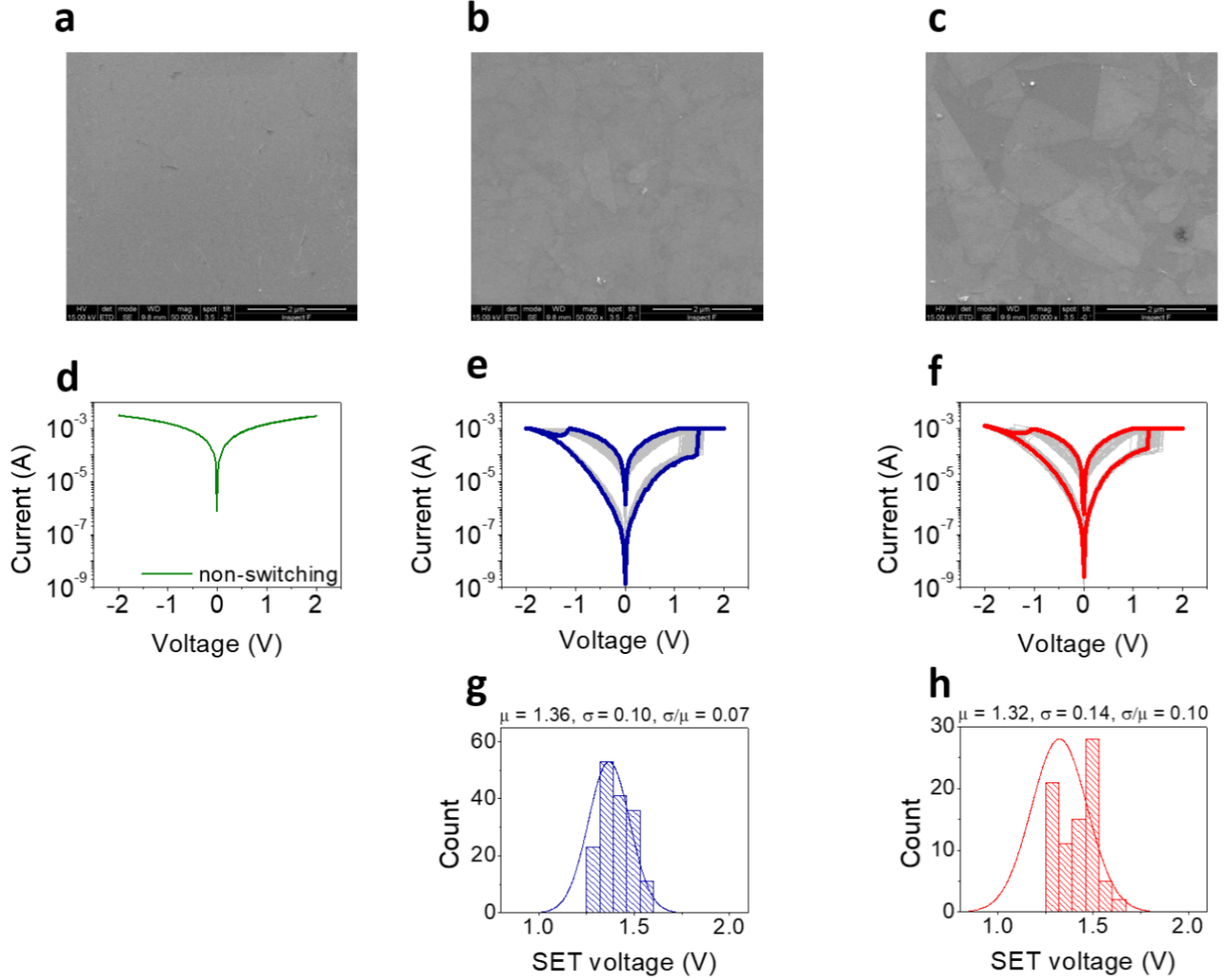


**Figure S4 | SEM images of 2D SNO PONs in (a) small, (b) medium, and (c) large sizes. *I-V* curves of 2D SNO PONs-based memristors fabricated using (d) small, (e) medium, and (f) large nanosheet sizes. Statistical analysis of the SET voltage variability for memristors fabricated using (g) medium and (h) large SNO PONs sizes.**


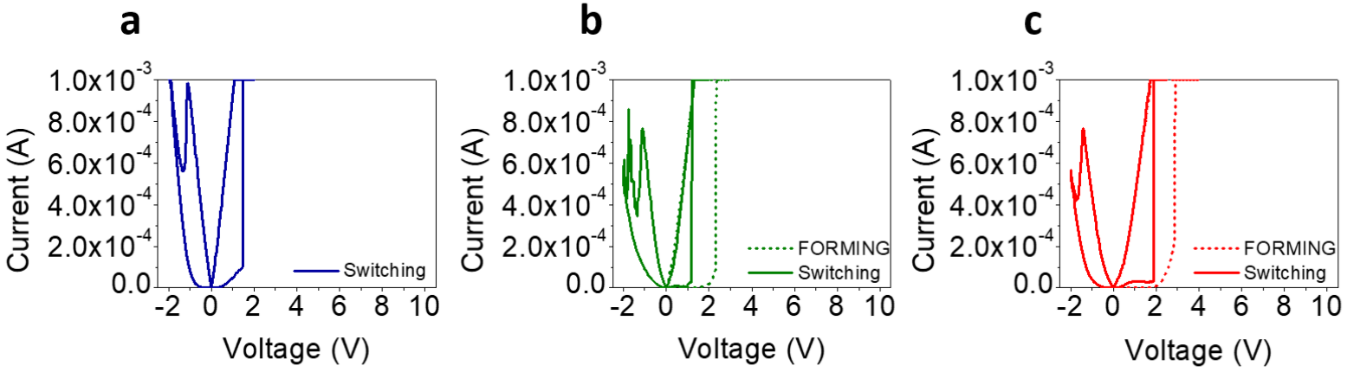


**Figure S5 | *I-V* curves of 2D SNO PONs-based memristors with varying numbers of SNO PONs layers: (a) 5-layer, (b) 7-layer, and (c) 10-layer devices.**


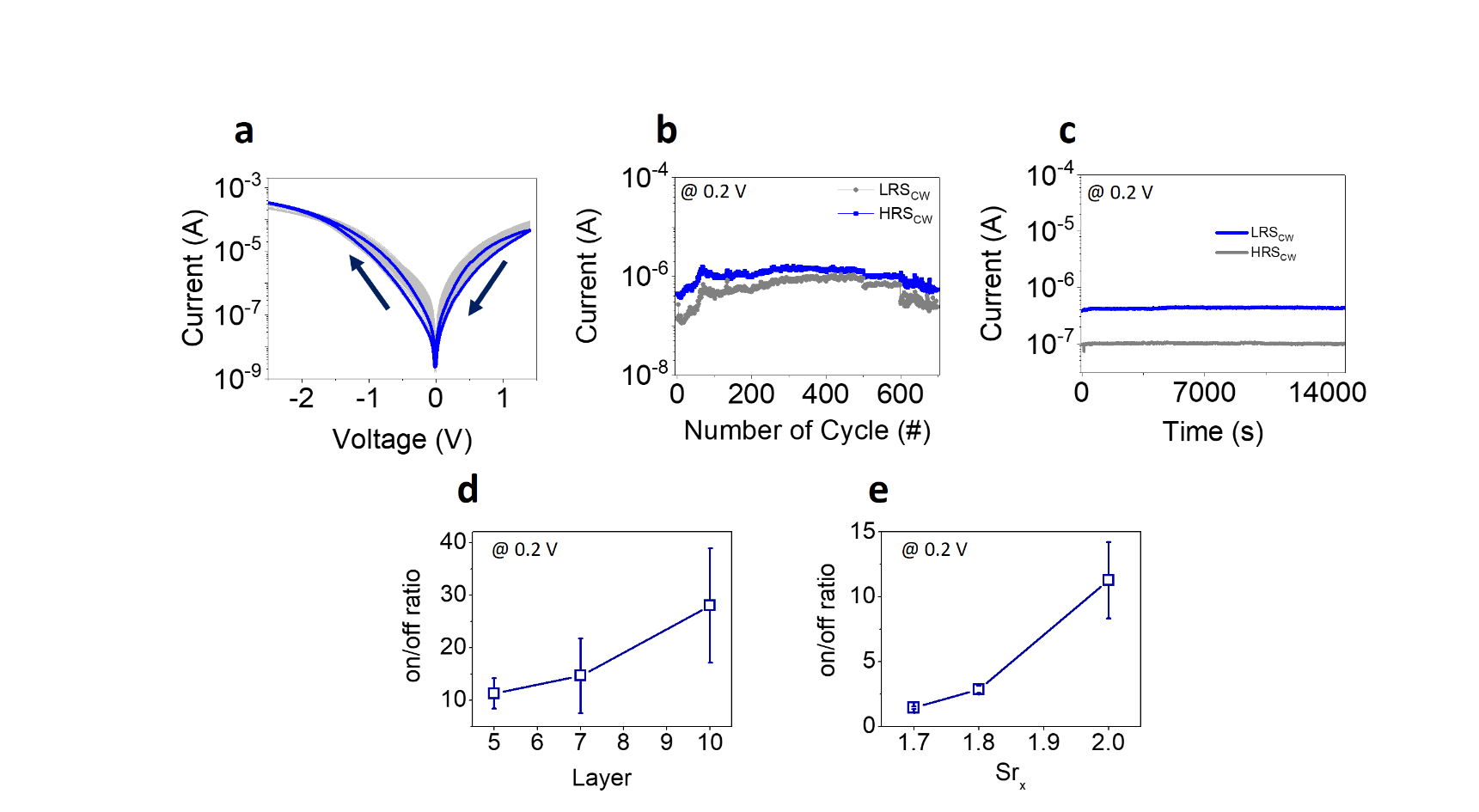
**Supporting Note 2:** CW RS behaviors in Au/Ti/2D SNO PONs/Pt memristor

**Figure S6 | (a) 700 representative *I-V* curves of a 2D SNO PONs-based memristor under CW RS. The blue arrows indicate the voltage sweep direction. (b) HRS and LRS current values obtained from the *I-V* curves shown in (a). (c) Retention characteristics of the LRS and HRS at a reading voltage of 0.2 V. (d) On/off ratio as a function of the number of 2D SNO PONs layers. (e) Modulation of the on/off ratio by tuning the Sr composition in the 2D SNO PONs.**

Figure S6a shows 700 representative clockwise (CW) RS cycles in a 2D SNO PONs-based memristor. The current values of HRS_CW_ and LRS_CW_ at a reading voltage of 0.2 V, derived from the repeatedly obtained *I-V* curves, are shown in Figure S6b, yielding an average on/off ratio of approximately 2. The mean current values for HRS_CW_ and LRS_CW_ are $6.25\times{10}^{-7} A$ and $1.09\times{10}^{-6} A$, respectively. Figure S6c demonstrates stable retention for both resistance states, confirming nonvolatile characteristics over 15,000 seconds.

The CW RS behavior in the Au/Ti/2D SNO PONs/Pt memristor is attributed to an exchange reaction between the Ti electrode and oxygen ions. This implies a strong dependence of RS behavior on the availability of mobile oxygen species. To investigate the correlation, memristors with varying layer numbers and systematically tuned Sr composition in the 2D SNO PONs were fabricated.

On/off resistance ratios at a reading voltage of 0.2 V were extracted from the *I–V* curves shown in Figure S7 for devices with varying SNO PON thicknesses and A-site compositions. As shown in Figure S6d, increasing the SNO PON thickness from 5 to 10 layers leads to a monotonic rise in the on/off ratio, indicating an expanded range of Ti-assisted redox reactions and formation of the thicker interfacial TiO_x_ layer, resulting in higher switching contrast. This behavior supports the cooperative TiO_x_ growth and V_O_ formation mechanism proposed in Figure 2b–2c. Figure S6e summarizes on/off ratios for devices with a fixed 5-layer thickness but varying Sr occupancy. A-site deficient devices contain pre-existing V_O_, thereby limiting oxygen extraction during the CW RESET process and suppressing the modulation between the LRS_CW_ and HRS_CW_ states. In contrast, stoichiometric SNO PON with a vacancy-poor lattice, allows for greater modulation of TiO_x_ thickness, thickening under CW RESET and thinning under CW SET, thus achieving significantly improved switching contrast. These thickness- and composition-dependent trends are fully consistent with the CW bipolar switching mechanism proposed in Figure 2b–2c.


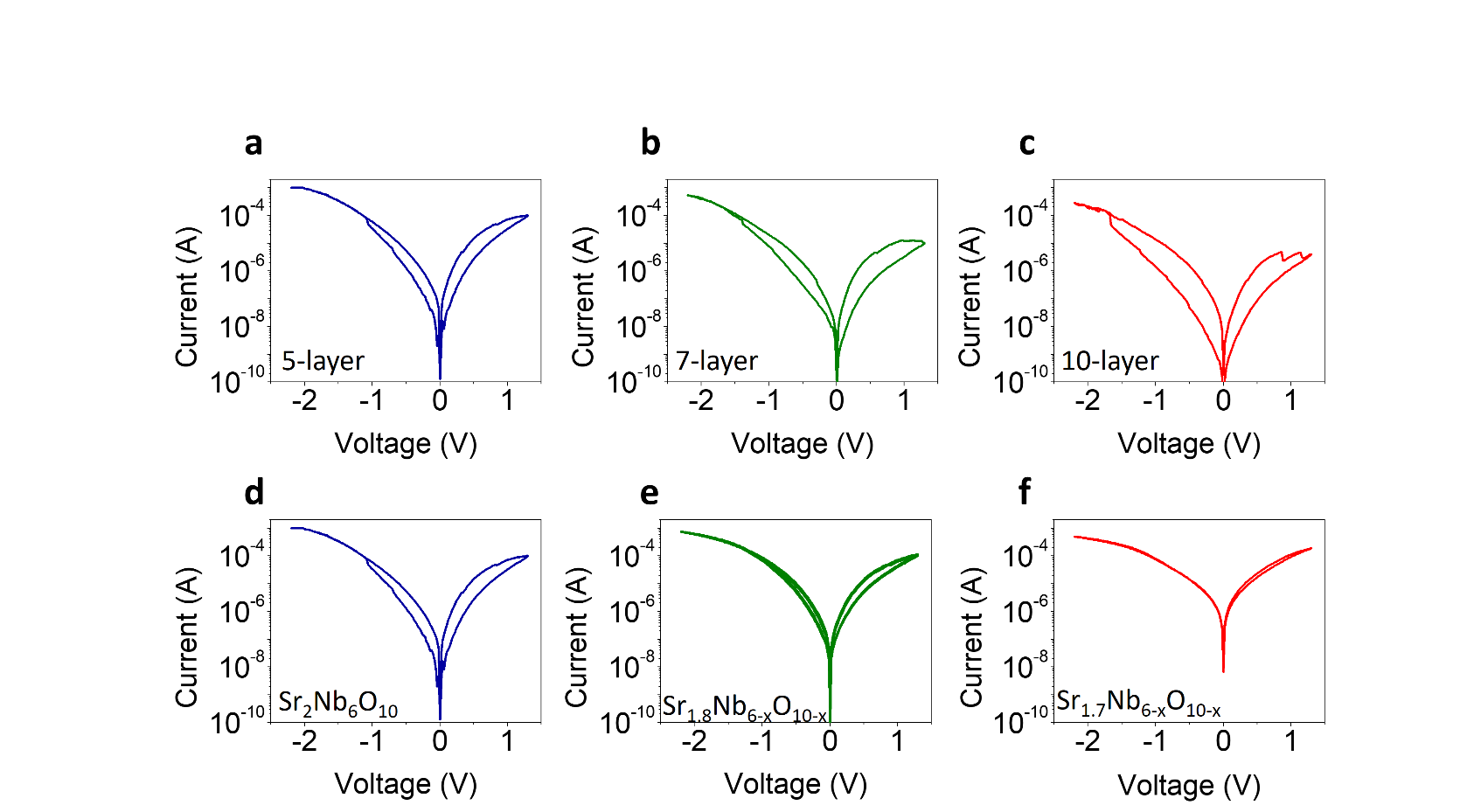


**Figure S7 | *I-V* curves of 2D SNO PONs-based memristors with varying layers of SNO PONs (a) 5-layer, (b) 7-layer, and (c) 10- layer and Sr composition (d) Sr_2_Nb_6_O_10_ (e) Sr_1.8_Nb_6_O_10_ (f) Sr_1.7_Nb_6-x_O_10-x_**

**_
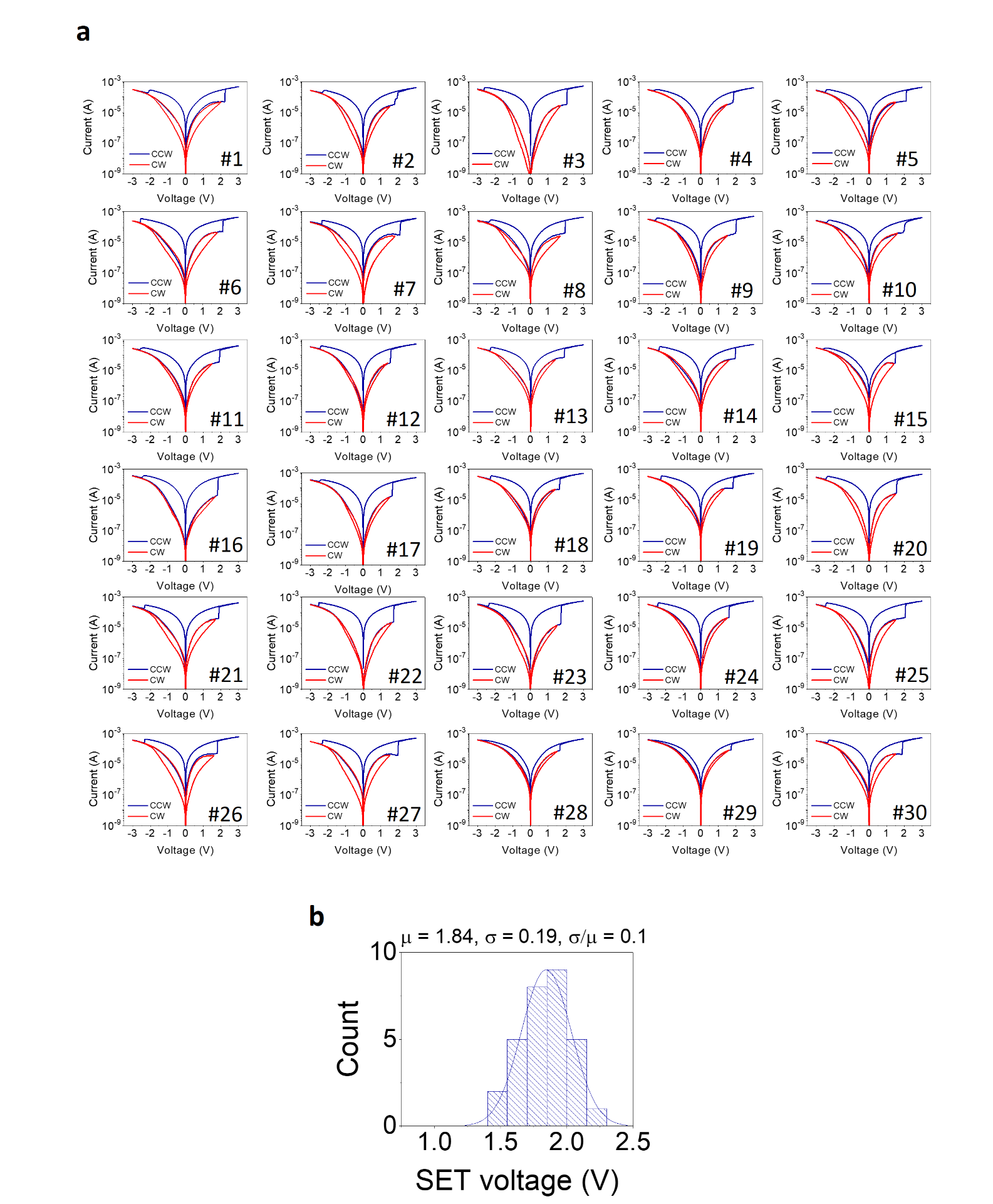
_**

**Figure S8 | (a) *I–V* characteristics of 30 individual 2D SNO PONs-based memristor devices. (b) Statistical analysis of the device-to-device variation in SET voltage extracted from (a).**

As shown in Figure S8a, *I–V* characteristics were measured from 30 individual memristor devices based on 2D SNO PONs. All devices exhibit reliable bipolar RS with well-defined CW and CCW modes, demonstrating excellent reproducibility across the device array. The statistical distribution of the set voltages, extracted from the repeated *I-V* measurements at a reading voltage of 0.2 V, is presented in Figure S8b. The mean ($\mu$) set voltage is 1.84 V, with a standard deviation ($\sigma$) of 0.19 V, resulting in a low device-to-device variability ($\sigma/\mu$) of 0.1. This high degree of uniformity suggests precise control over the switching layer and the consistent formation of conductive filaments across the fabricated devices.


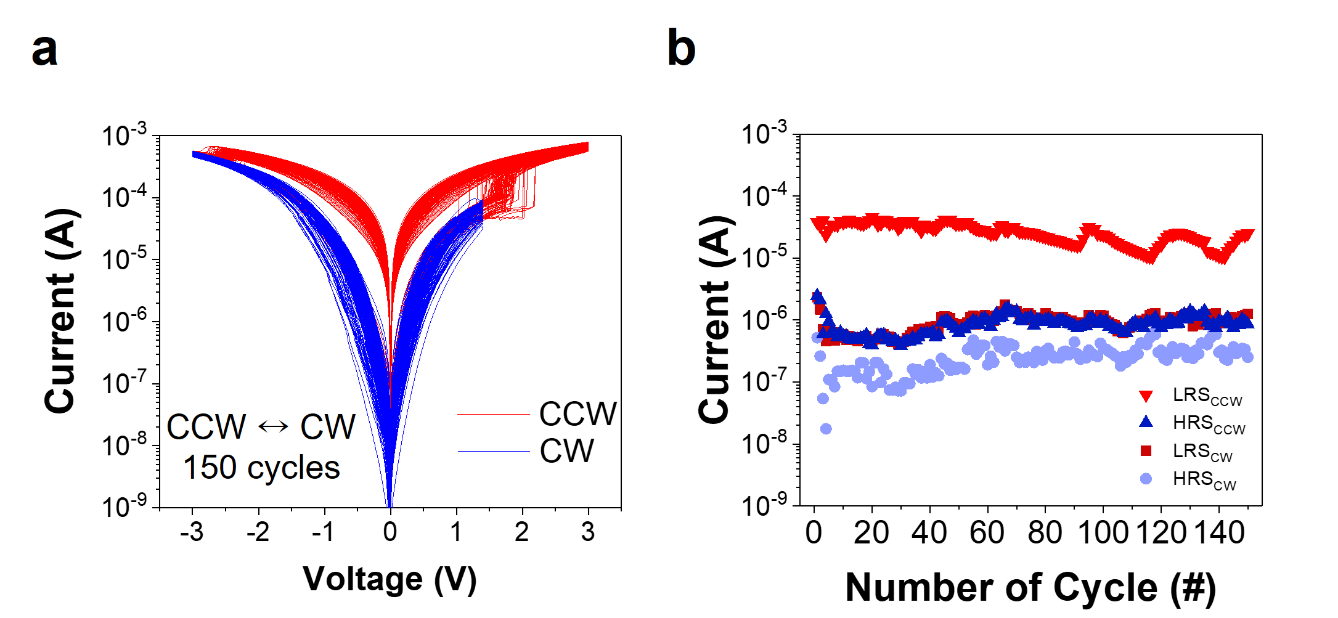


**Figure S9 | (a) Reproducible CW↔CCW mode-switching over 150 cycles of 2D SNO PONs-based memristor devices. (b) Statistical analysis of the cycle-to-cycle variation in all four resistance states.**

Reproducible CW↔CCW mode-switching over 150 cycles, while exhibiting σ/μ of 32.4 %, 46.0 %, 34.7 %, and 34.8 %) across the four read states (LRS_CW_, HRS_CW_, HRS_CCW_, and LRS_CCW_). Despite this variability, the four states remain clearly separable under a 0.2 V read (average on/off ≈ 20 for CCW and ≈ 2 for CW), indicating functionally stable and reversible Ti-assisted redox switching.


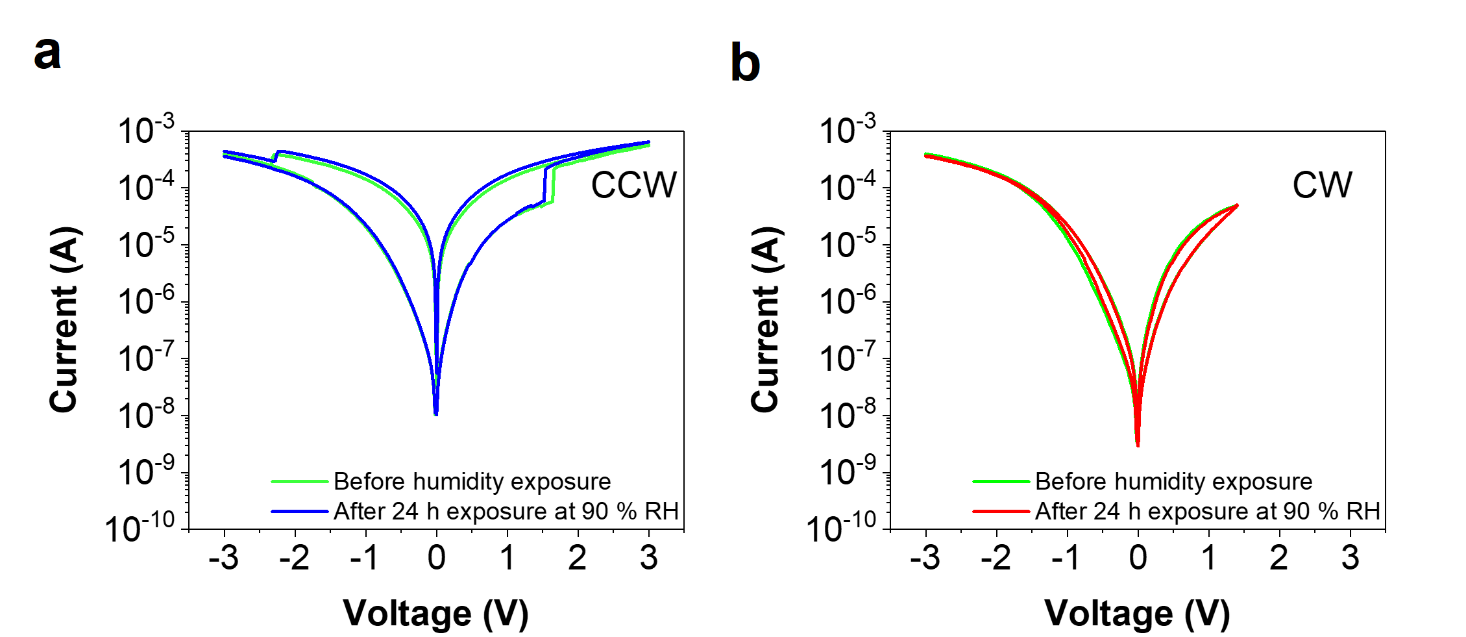


**Figure S10 | Humidity stability of the 2D SNO PONs memristor. (a) CCW and (b) CW modes of the Au/Ti/2D SNO PONs/Pt memristor measured before and after 24 hours of humidity exposure at 90 % relative humidity (RH) and 25 °C.**

Humidity exposure tests were carried out to evaluate the environmental reliability of the 2D SNO PONs memristor. Devices were placed in a sealed acrylic chamber maintained at 25 °C and 90 % relative humidity (RH) for 24 h.The 90 % RH condition was achieved using a saturated potassium nitrate (KNO₃) solution, following the ASTM E104-02 standard, and the humidity level was monitored with a digital hygrometer throughout the exposure period. After exposure, the *I–V* characteristics were re-measured under ambient conditions. As shown in Figure S10, the switching behavior and current levels remain virtually unchanged compared to those measured before humidity exposure. This result confirms that the Au/Ti/2D SNO PONs/Pt memristor maintains its bipolar switching performance and structural stability even after prolonged exposure to high humidity, demonstrating excellent environmental robustness suitable for long-term device operation.

**
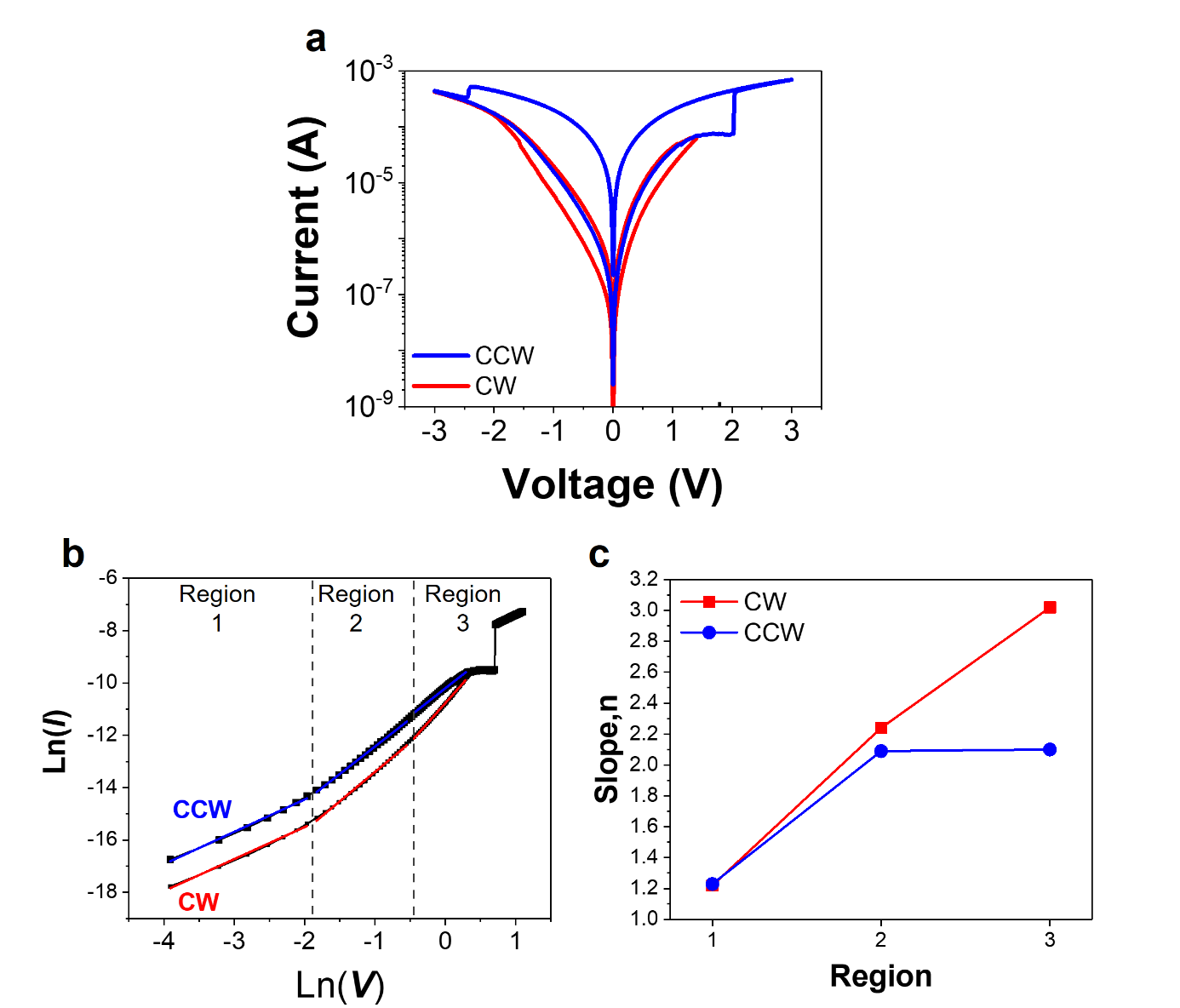
**

**Figure S11 | Space-charge-limited current (SCLC) analysis of the *I–V* characteristics for both clockwise (CW) and counterclockwise (CCW) switching modes. (a) Measured *I–V* characteristics illustrating distinct current responses under CW and CCW bias sweeps. (b) Corresponding log–log plots of current versus voltage, exhibiting clear slope transitions that reveal different conduction regimes. (c) Extracted slope (n) values from Figure S11b**.

We performed space-charge-limited current (SCLC) fitting of the measured *I–V* characteristics for both switching modes. In the CW mode, the *log(I)–log(V*) plot exhibits three linear regions with slopes of *n* ≈ 1.22 (0.02–0.15 V), *n* ≈ 2.24 (0.15–0.7 V), and *n* ≈ 3.02 (0.7–1.3 V). This progressive increase of *n* indicates a transition from Ohmic conduction to trap-controlled and trap-filled SCLC, signifying barrier-limited charge injection and interface-controlled redox conduction at the Ti/SNO boundary^8-10^.

In contrast, the CCW mode shows smaller *n* values (*n* ≈ 1.23–2.1) for the same voltage ranges), suggesting that the current flow is governed by localized oxygen-vacancy filaments forming quasi-metallic conduction channels. The weaker voltage dependence in CCW implies that the transport is dominated by bulk-limited, field-enhanced conduction rather than interface modulation^11^.

Therefore, the SCLC analysis provides quantitative electrical evidence for the coexistence of two distinct mechanisms: the CW mode governed by interfacial barrier-controlled SCLC, and the CCW mode dominated by filamentary and bulk-limited conduction.

**
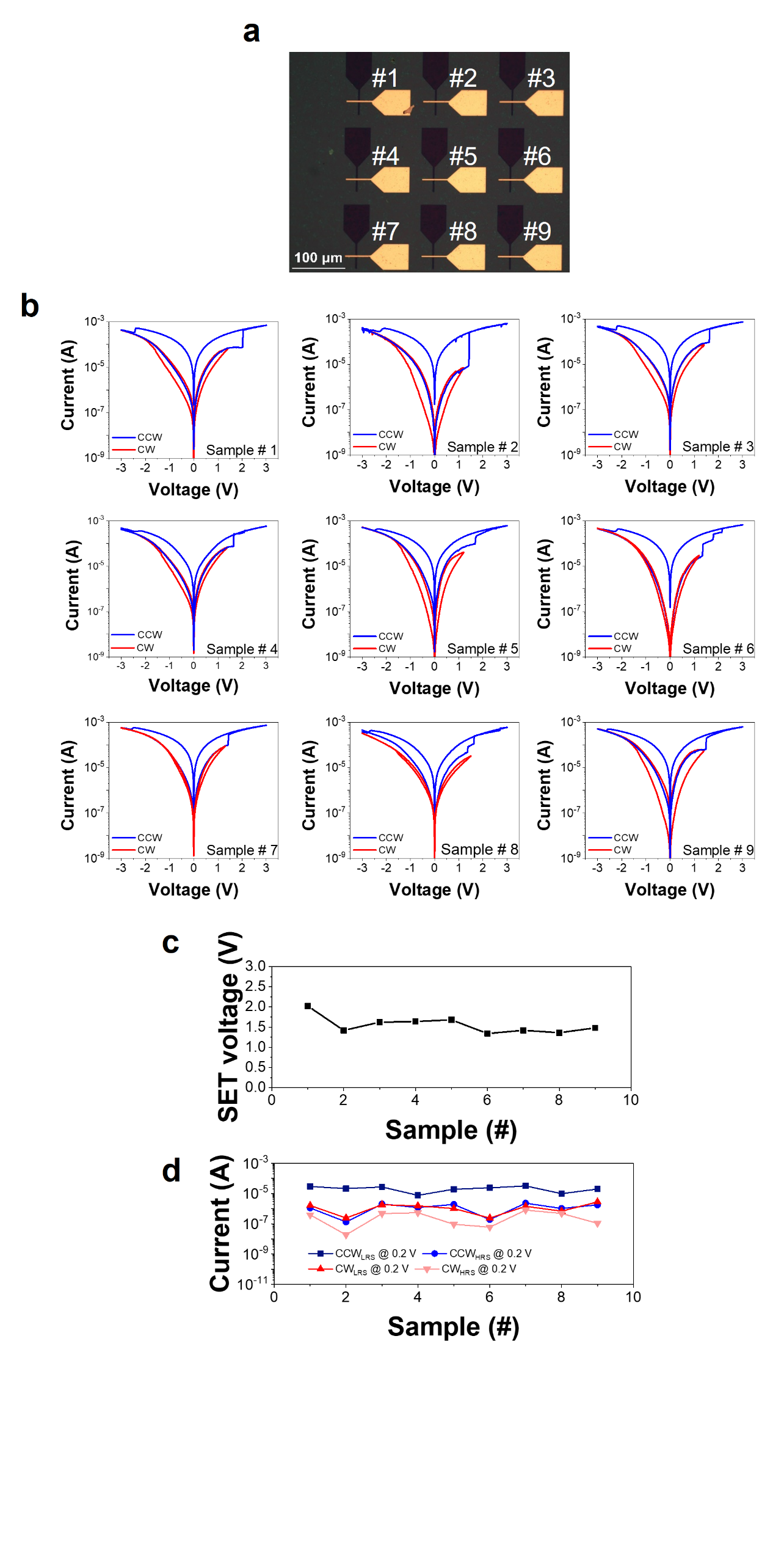
**

**Figure S12 | Optical image and statistical analysis of the fabricated 3 × 3 Au/Ti/2D SNO PONs/Pt memristor array. (a) Optical image of the 3 × 3 array corresponds to the fabricated cross-point device configuration, where each memristive cell consists of a single top electrode and a single bottom electrode. (b) *I-V* curves of all nine devices. (c) Set voltage and (d) HRS/LRS currents at a read bias of 0.2 V, measured for both CW and CCW modes across all nine devices, as extracted from Figure S11 (b).**

The 3 × 3 array corresponds to an actual fabricated device array in a cross-point configuration, where each memristive cell consists of a single bottom electrode and a single top electrode connected at one junction. Nine such devices are arranged in a 3 × 3 grid on the same substrate. An optical microscope image of the fabricated array shows uniform electrode morphology and spatially consistent device spacing across the wafer (Figure S12a).

To evaluate the uniformity of the fabricated array, the set voltage and HRS/LRS currents at a read bias of 0.2 V were obtained for both CW and CCW modes from the *I-V* curves of all nine devices shown in Figure S12b. Figures S12c and S12d reveal that the set voltages ranges from 1.34 V to 2.02 V with a mean of 1.53 V and a relative standard deviation (σ/μ) of 3.8 %, while both CW and CCW LRS/HRS currents exhibit σ/μ < 4 %. These results demonstrate excellent device-to-device uniformity and reproducibility of interfacial and filamentary switching behaviors within the 3×3 array.

**
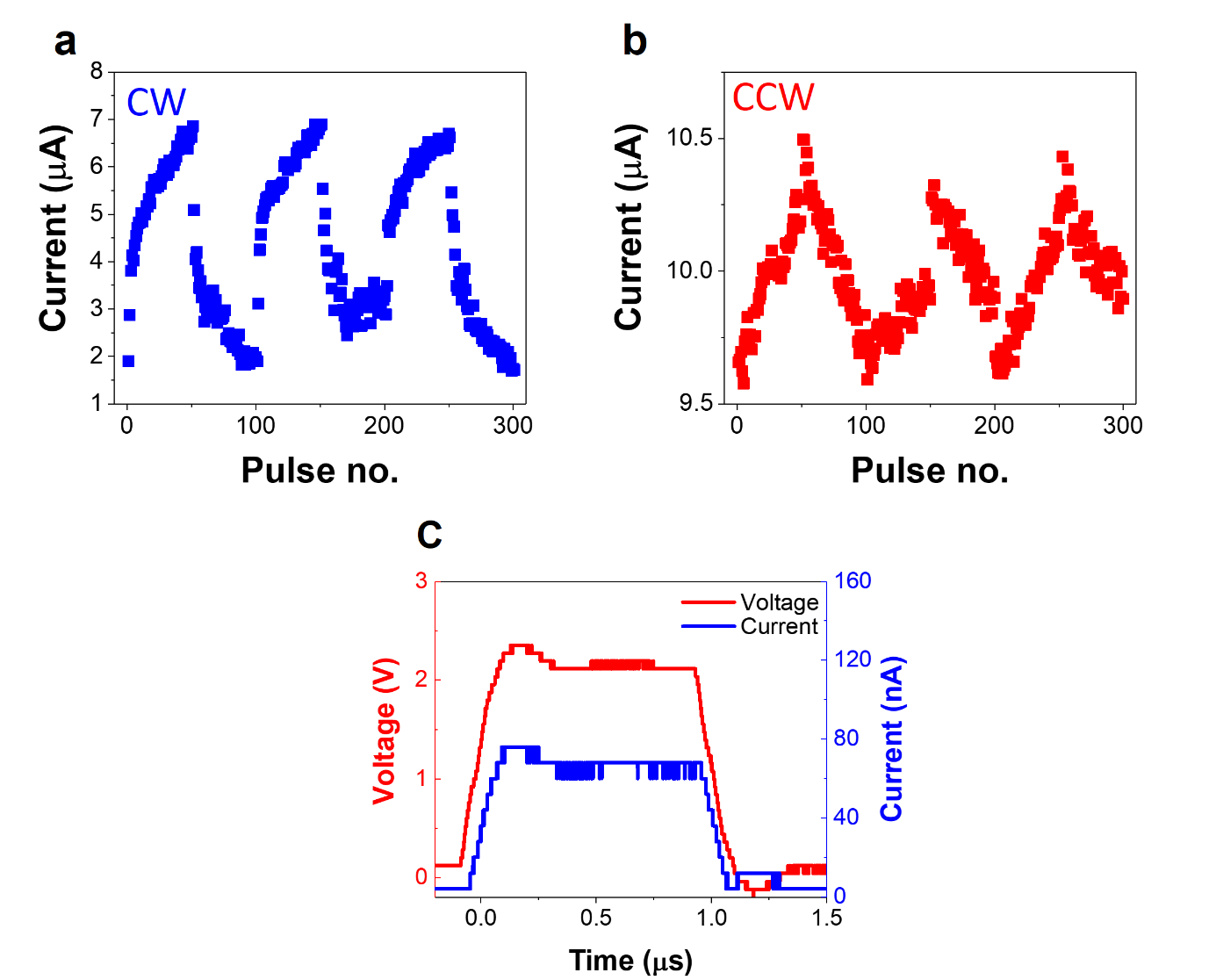
**

**Figure S13 | 1 Microsecond synaptic modulation of 2D SNO PONs memristor. (a) Synpatic updates for CW and CCW mode. (b) Energy consumption per synaptic update.**

In the CW mode (interfacial switching), 50 positive pulses of 2 V and 1 µs duration were applied for depression, followed by 50 negative pulses of -3 V and 1 µs for potentiation, with readings taken at 0.2 V. A gradual and reversible increase or decrease in conductance was observed, confirming stable interface-type weight modulation at the microsecond scale. In the CCW mode (filamentary switching), 50 positive pulses of 3.5 V and 1 µs were applied for potentiation, followed by 50 negative pulses of -4 V and 1 µs for depression, with readings taken again at 0.2 V. The current increased and decreased progressively in small increments, evidencing partial filament formation and rupture dynamics suitable for analog synaptic behavior. Both CW and CCW switching events occurred reliably within a 1 µs pulse width, confirming that conductance updates can be triggered within sub-microsecond timescales.

To verify the energy efficiency of the 2D SNO PONs memristor, ultrafast pulse measurements were performed using a Keithley 4200 semiconductor characterization system (as a pulse generator) and a Tektronix oscilloscope. Single voltage pulse (2 V and 1 µs) was applied to the top electrode while grounding the bottom electrode to monitor the overall resistive switching dynamics. The input voltage (Channel 1) and the transmitted signal through the device (Channel 2) were simultaneously recorded to calculate the instantaneous power. The energy per switching event, obtained by integrating the instantaneous power ($E=\int V(t)\text{ }I(t)\text{ }\mathrm{dt}$), was approximately 0.14 pJ per synaptic update. For comparison, recent studies have reported energy consumptions of ≈ 0.36 pJ in ferroelectric optoelectronic synaptic devices^12^ and ≈ 0.1 pJ per write event in RRAM-based neuromorphic systems^13^. In addition, large-scale dendritic RRAM networks have achieved energy-efficient temporal processing comparable to our results^14^. These comparisons confirm that the proposed 2D SNO PON memristor operates within the same ultralow-energy regime as state-of-the-art synaptic devices.

**Supplementary Note 3: Incorporation of hardware-level effects into ReSuMe simulation**

Figures S14-S16 presents additional simulations incorporating hardware-level effects such as device non-idealities, variability, and sneak paths. These simulations were performed to evaluate whether the proposed ReSuMe-based learning scheme can operate reliably under realistic hardware conditions.


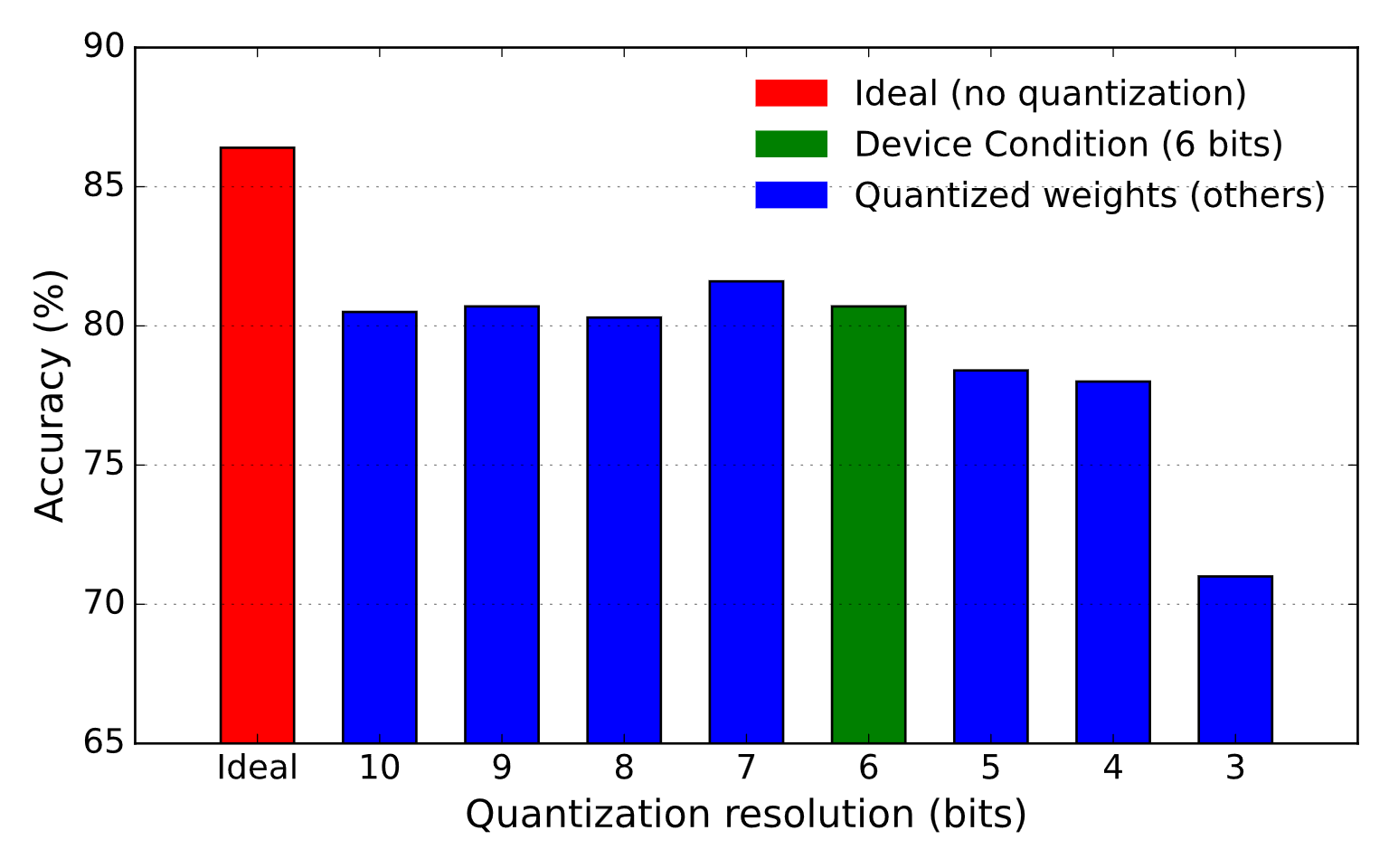


**Figure S14 | Impact of non-idealities (synaptic quantization resolution) on ReSuMe simulation.**

To evaluate the influence of finite conductance quantization on network performance, we simulated the ReSuMe-based spike learning using quantized weight levels between $G_{min}$ and $G_{max}$. The 6-bit resolution corresponds to the experimentally measured number of stable conductance states in our device, while other resolutions represent hypothetical cases of higher or lower quantization. The results demonstrate that the classification accuracy remains robust (> 80%) for 6-bit resolution and shows a gradual decline for lower bit precision, indicating that the intrinsic device quantization is sufficient for efficient learning within the proposed spike-based framework.


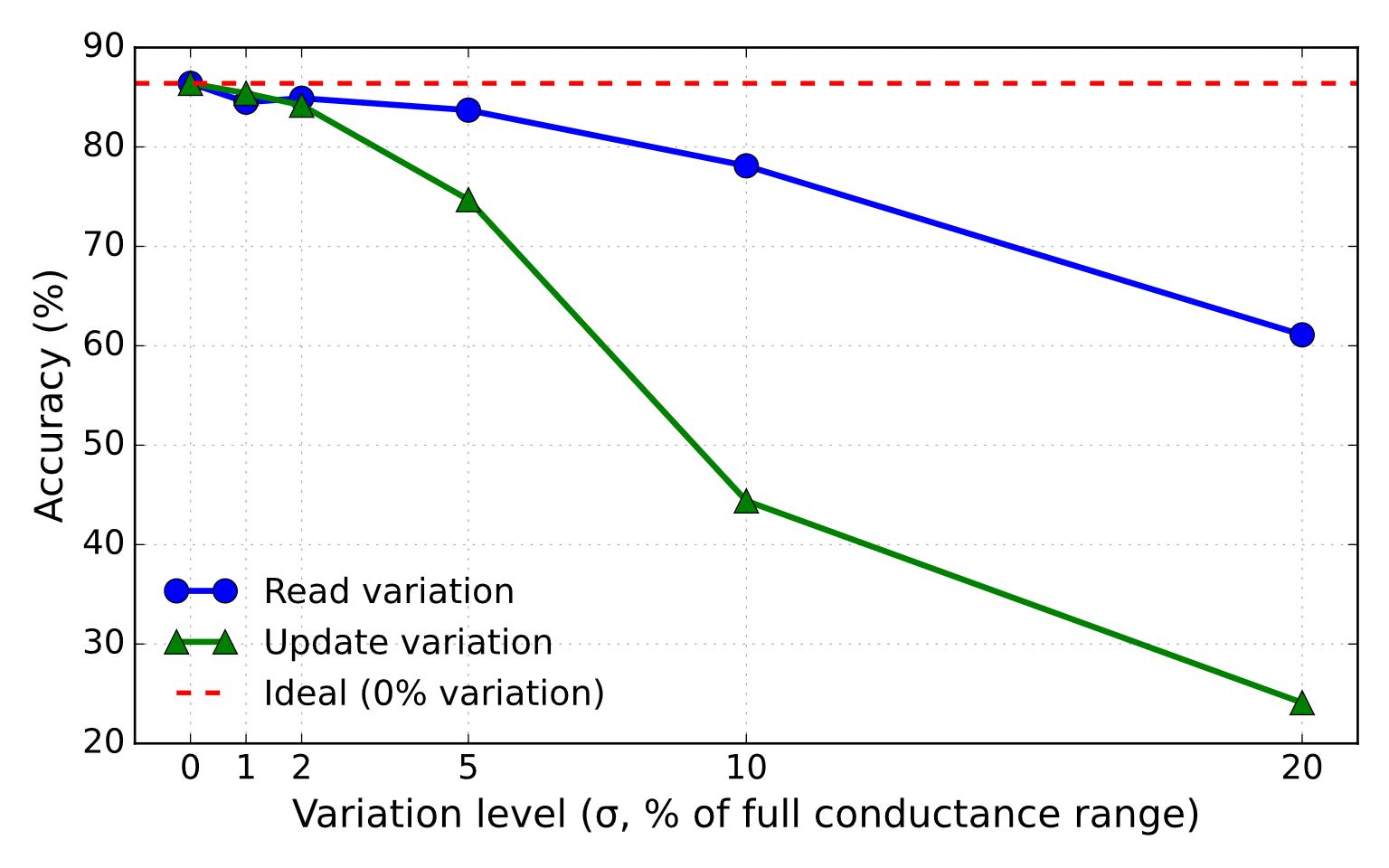


**Figure S15 | Impact of device variability on ReSuMe simulation.**

We modeled two primary types of conductance fluctuations: (i) read variation, representing random noise during readout operations, and (ii) update variation, representing stochastic weight changes during potentiation and depression events. As shown in Figure S9, the network maintains over 80% accuracy under realistic variation levels of a few percent, indicating strong tolerance to intrinsic read noise. However, performance degradation becomes evident beyond 10% variation, particularly for update variability, which directly affects the learning process. These results highlight that precise and stable conductance modulation is critical to maintaining learning reliability in memristor-based crossbar systems.


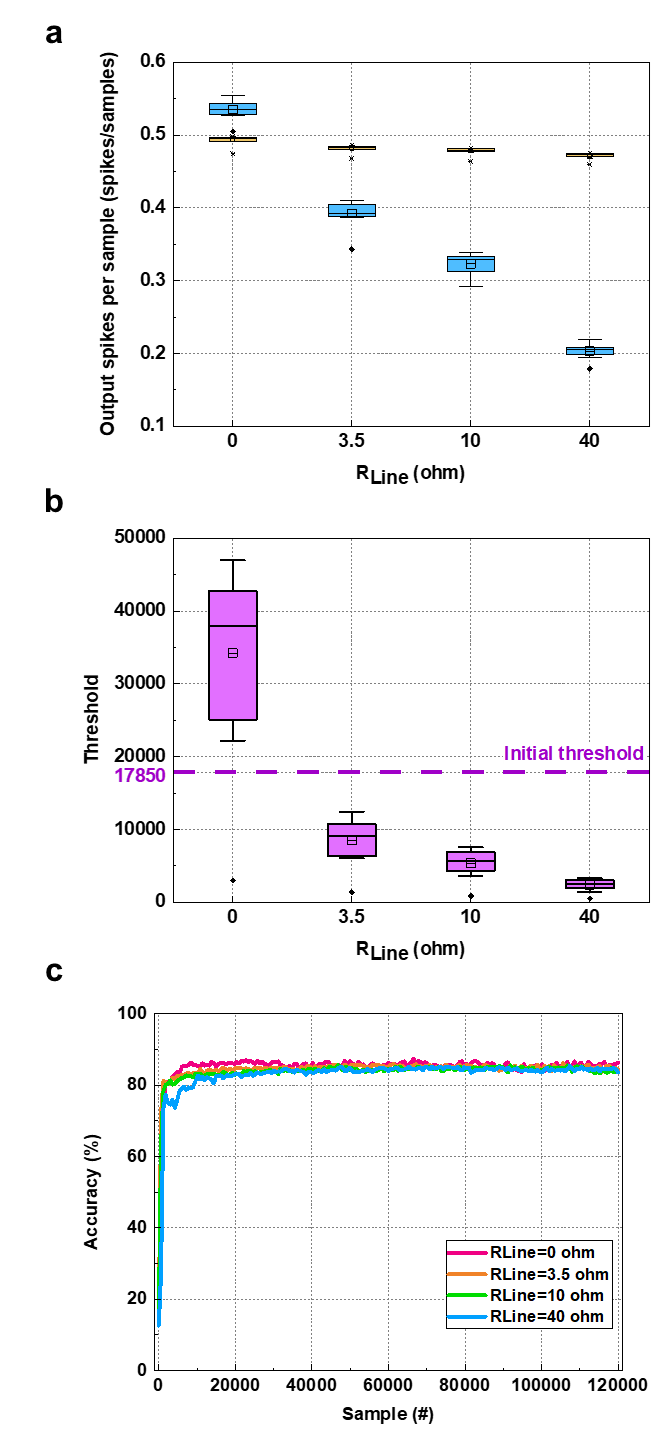


**Figure S16 | Impact of sneak path current on ReSuMe simulation. (a) Distribution of spikes generated by the 10 output neurons after 5,000 training samples (blue box plot) and 120,000 training samples (yellow box plot). (b) Distribution of threshold value after ReSuMe simulation with and without line resistance. (c) Accuracy results of the ReSuMe simulation.**

To evaluate ReSuMe algorithm under sneak path conditions, we followed the procedure below. (1) We modeled a $784\times10$ crossbar array based on our device with random weight under two conditions: an ideal case without line resistance $(R_{Line}=0 \Omega)$ and a real case with line resistance $(R_{Line}=3.5, 10, a\mathrm{nd} 40 \Omega)$^15^. (2) For each condition, we applied 50,000 input spikes, generated by a Poisson process, to 100 randomly selected MNIST images and computed the output current $(I_{out})$ for every spike. (3) Using the two sets of $I_{out}$, we computed the sample-wise ratio between conditions, then estimated the mean $\mu$ and standard deviation $\sigma$, and modeled the distribution as $N(\mu, \sigma^{2})$ (Table 1). (4) In the ReSuMe algorithm, we drew a $scaling factor (s)\sim N(\mu, \sigma^{2})$ and incorporated it into the output neuron’s membrane potential calculation whenever an output current was stored. We clipped it as $s^{'}=max(s, 0)$ because the distribution can yield negative values^16^.

|  | μ | σ |
| --- | --- | --- |
| 3.5 Ω | 0.23278 | 0.16201 |
| 10 Ω | 0.12374 | 0.16550 |
| 40 Ω | 0.0472 | 0.0894 |

**Supplementary Table S1 | Mean and standard deviation of the output current ratio for a real case relative to an ideal case.**

Figure S16a shows the distribution of output spikes from the ten output neurons after 5,000 (blue) and 120,000 (yellow) training samples. At the beginning of training, the presence of sneak paths reduced the number of output spikes by approximately 27%, 40%, and 62% for $R_{Line}=3.5, 10 \mathrm{and} 40\Omega$ respectively, compared with the ideal case. This reduction weakened long-term depression (LTD) relative to potentiation, disturbing the LTP/LTD balance and leading to a temporary drop in accuracy.

To stabilize the learning dynamics, an adaptive threshold (homeostatic) mechanism was employed^17-20^. In this model, each output neuron dynamically adjusts its firing threshold according to recent activity—decreasing when the spike count falls below a homeostatic target and increasing when it exceeds that target.

This proportional update, implemented in the simulator, reflects a biologically observed homeostatic process^17,18^ and prevents saturation by maintaining balanced excitability. Consequently, the adaptive threshold compensates for reduced membrane potential under sneak-path and line-resistance conditions. As shown in Figure S16c, this mechanism effectively restored stable spike activity, limiting accuracy degradation to less than ~3%.

In the simulator, the threshold was updated proportionally to the deviation between the actual and desired firing rates, following a homeostatic update rule:

$$V_{\text{TH}}\left( t+1 \right)=V_{\text{TH}}\left( t \right)+r\cdot\left( N_{\text{spikes}}-N_{\text{target}} \right)\cdot V_{\text{TH}}\left( t \right)$$

and its temporal decay:

$$V_{\text{TH}}\left( t \right)=V_{\text{TH}}\left( t \right)\cdot\exp\left( -1/\tau_{\text{th}} \right)$$

This rule corresponds to functions in the simulation code, ensuring a biologically inspired self-regulation of excitability that maintains stable firing activity under varying hardware conditions. Overall, the adaptive threshold mechanism provides effective homeostatic compensation, maintaining sufficient output spike activity for stable learning even in the presence of strong sneak-path and line-resistance effects.

**References**

1. B. Tang, H. Veluri, Y. Li, Z. G. Yu, M. Waqar, J. F. Leong, M. Sivan, E. Zamburg, Y.-W. Zhang, J. Wang, and A. V.-Y. Thean, “Wafer-Scale Solution-Processed 2D Material Analog Resistive Memory Array for Memory-Based Computing,” *Nature Communications* 13 (2022): 3037.
2. Y. Shi, S. D. Ha, Y. Zhou, F. Schoofs, D. J. Groenendijk, J. Hu, Y. Li, Y. Yi, X. Yu, C. Li, S. Yuan, Z. Wang, Z. Guo, J. Shen, H. Yuan, L. Sun, J. Jia, and H. Wang, “Electronic Synapses Made of Layered Two-Dimensional Materials,” *Nature Electronics* 1 (2018): 458.
3. S. Chen, R. Mahmoodi, Y. Shi, C. Mahata, B. Yuan, X. Liang, C. Wen, F. Hui, D. Akinwande, D. Strukov, and M. Lanza, “Wafer-Scale Integration of Two-Dimensional Materials in High-Density Memristive Crossbar Arrays for Artificial Neural Networks,” *Nature Electronics* 3 (2020): 638.
4. C. Pan, S. Wang, J. Li, J. Yang, and F. Miao, “Coexistence of Grain-Boundaries-Assisted Bipolar and Threshold Resistive Switching in Multilayer Hexagonal Boron Nitride,” *Advanced Functional Materials* 27 (2017): 1604811.
5. J. Xie, S. Afshari, and I. Sanchez Esqueda, “Hexagonal Boron Nitride (h-BN) Memristor Arrays for Analog-Based Machine Learning Hardware,” *npj 2D Materials and Applications* 6 (2022): 50.
6. Y. Shen, Y. Li, M. Lanza, F. Hui, R. Zhang, J. Yu, X. Liang, Z. Zhang, B. Yuan, H. Yuan, J. Wu, and H. Wang, “Variability and Yield in h-BN-Based Memristive Circuits: The Role of Each Type of Defect,” *Advanced Materials* 33 (2021): 2103656.
7. R. T. Tung, “The Physics and Chemistry of the Schottky Barrier Height,” *Applied Physics Reviews* 1 (2014): 011304.
8. Z. Chiguvare, “Electrical and Optical Characterization of Bulk Heterojunction Polymer- Fullerene Solar Cells,” Ph.D Thesis, Oldenburg University (2005).
9. K. Karmakar, A. Roy, S. Dhibar, S. Majumder, S. Bhattacharjee, S. K. M. Rahaman, R. Saha, P. Chatterjee, S. J. Ray, and B. Saha, “Exploration of a Wide Bandgap Semiconducting Supramolecular Mg(II) Metallohydrogel Derived from an Aliphatic Amine: A Robust Resistive Switching Framework for Brain-Inspired Computing,” [*Scientific Reports*](https://www.nature.com/srep)  13 (2023): 22318.
10. A. Sawa, T. Fujii, M. Kawasaki and Y. Tokura, “Hysteretic Current–Voltage Characteristics and Resistance Switching at a Rectifying Ti/Pr_0.7_Ca_0.3_MnO_3_ Interface,” *Applied Physics Letters* 85 (2004): 4073-4075.
11. Y. B. Zhu1, K. Zheng, X. Wu and L. K. Ang, “Enhanced Stability of Filament-Type Resistive Switching by Interface Engineering,” *Scientific Reports* 7 (2017): 43664.
12. Z. Shang, L. Liu, G. Wang, H. Xu, Y. Cui, J. Deng, Z. Lou, Y. Yan, J. Deng, S.-T. Han, T. Zhai, X. Wang, L. Wang and X. Wang, “Ferroelectric Polarization Enhanced Optoelectronic Synaptic Response of a CuInP₂S₆ Transistor Structure,” *ACS Nano* 18 (2024): 30530-30539.
13. K. U. Mohanan, “Resistive Switching Devices for Neuromorphic Computing: From Foundations to Chip Level Innovations,” *Nanomaterials* 14 (2024): 527.
14. S. D’Agostino, F. Moro, T. Torchet, Y. Demirağ, L. Grenouillet, N. Castellani, G. Indiveri, E. Vianello, M. Payvand, “DenRAM: Neuromorphic Dendritic Architecture with RRAM for Efficient Temporal Processing with Delay,” *Nature Communications* 15 (2024): 3446.
15. J. E. Kim, S. Hu, J. Y. Kwon, S. Y. Chun, K. Soh, H. Yun, S-H. Baek, S. Nahm, Y. J. Jeong and J. H. Yoon, “Cluster-Type Conductive Path-Based Selector-Less 1R Memristor Array for Spiking Neural Networks,” *Nano Energy* 140 (2025): 110983.
16. I. Chakraborty, M. Fayez Ali, D. E. Kim, A. Ankit and K. Roy, “GENIEx: A Generalized Approach to Emulating Non-Ideality in Memristive Xbars Using Neural Networks,” *Proceedings of the 57th ACM/IEEE Design Automation Conference (DAC)*, doi:10.1109/DAC18072.2020.9218688.
17. R. Azouz and C. M. Gray, “Dynamic Spike Threshold Reveals a Mechanism for Synaptic Coincidence Detection in Cortical Neurons In Vivo,” *Proceedings of the National Academy of Sciences* 97 (2000): 8110–8115.
18. J. Platkiewicz and R. Brette, “A Threshold Equation for Action Potential Initiation,” *PLoS Computational Biology* 6 (2010): e1000850.
19. T. Kim, S. Hu, J. Kim, J-Y. Kwak, J. Park, S. Lee, I. Kim, J-K. Park and Y. Jeong, “Spiking Neural Network (SNN) with Memristor Synapses Having Non-Linear Weight Update,” *Frontiers in Computational Neuroscience* 15 (2021): 646125.
20. P. U. Diehl and M. Cook, “Unsupervised Learning of Digit Recognition Using Spike-Timing-Dependent Plasticity,” *Frontiers in Computational Neuroscience* 9 (2015): 99.
